# 2DB: a Proteomics database for storage, analysis, presentation, and retrieval of information from mass spectrometric experiments

**DOI:** 10.1186/1471-2105-9-302

**Published:** 2008-07-07

**Authors:** Jens Allmer, Sebastian Kuhlgert, Michael Hippler

**Affiliations:** 1 Institute for Plant Biochemistry and Biotechnology, University of Münster, Hindenburgplatz 55, Münster, Germany; 2 Computer Science, Izmir University of Economics, Sakarya Caddesi 156, Balçova, Izmir, Turkey

## Abstract

**Background:**

The amount of information stemming from proteomics experiments involving (multi dimensional) separation techniques, mass spectrometric analysis, and computational analysis is ever-increasing. Data from such an experimental workflow needs to be captured, related and analyzed. Biological experiments within this scope produce heterogenic data ranging from pictures of one or two-dimensional protein maps and spectra recorded by tandem mass spectrometry to text-based identifications made by algorithms which analyze these spectra. Additionally, peptide and corresponding protein information needs to be displayed.

**Results:**

In order to handle the large amount of data from computational processing of mass spectrometric experiments, automatic import scripts are available and the necessity for manual input to the database has been minimized. Information is in a generic format which abstracts from specific software tools typically used in such an experimental workflow. The software is therefore capable of storing and cross analysing results from many algorithms. A novel feature and a focus of this database is to facilitate protein identification by using peptides identified from mass spectrometry and link this information directly to respective protein maps. Additionally, our application employs spectral counting for quantitative presentation of the data. All information can be linked to hot spots on images to place the results into an experimental context. A summary of identified proteins, containing all relevant information per hot spot, is automatically generated, usually upon either a change in the underlying protein models or due to newly imported identifications. The supporting information for this report can be accessed in multiple ways using the user interface provided by the application.

**Conclusion:**

We present a proteomics database which aims to greatly reduce evaluation time of results from mass spectrometric experiments and enhance result quality by allowing consistent data handling. Import functionality, automatic protein detection, and summary creation act together to facilitate data analysis. In addition, supporting information for these findings is readily accessible via the graphical user interface provided. The database schema and the implementation, which can easily be installed on virtually any server, can be downloaded in the form of a compressed file from our project webpage.

## Background

One major challenge in proteomics is the identification of proteins within a specific experimental context. The methods employed in these fields are numerous. Although multi-dimensional liquid chromatography (LC) methods coupled to mass spectrometry (MS) are advancing [1-3], two-dimensional gel electrophoresis combined with MS is still a major method for proteome analysis [4].

MS is currently the tool of choice for peptide and protein identification [5]. For this, a bottom-up strategy is most widely employed in MS [6]. Using this method, proteins are first cleaved into peptides by a protease (usually trypsin). These peptides are then analyzed using MS or tandem MS. The resulting tandem mass spectra are typically submitted to computational analysis by algorithms which correlate spectra to entries in multiple amino acid sequence databases. Although there are numerous software tools which can perform this mapping, the two most widespread are Sequest [7] and Mascot [8] which currently represent the industry standard [9]. The results of this analysis are amino acid sequences which have been successfully mapped to MS/MS spectra.

The set of resulting peptides from this analysis can be used to identify proteins. A protein with two or more supporting peptides is widely accepted as a confident identification [10]. A protein with a single supporting peptide can be accepted as a confident identification when de novo amino acid sequencing and correlation analysis together give supportive evidence [11].

As can be deduced from the simplified view of proteomics above, data from proteomics experiments are extremely heterogeneous. The challenge in proteomics is to integrate all this data into one data warehouse enabling searches and creating relationships across different topics. A part of this enormous task is addressed in this paper. Our initial interest was the identification of proteins from experiments which can be represented as pictures containing specific areas of interest (hot spots) which were examined by MS/MS with subsequent computational analysis. There is, however, no limitation imposed by this and experiments do not need pictorial representation although it enhances their presentation and usability.

It is important to connect areas of interest in a picture (i.e. spots on a 2-DE gel) to results from subsequent analysis. To achieve this, it is necessary to define these spots, and for this purpose a software tool which is integrated into the 2DB application (see Additional file 1 and [12]) is provided, directly allowing definition of areas on the picture while enabling the specification of additional information for each.

The bottom-up strategy employed in mass spectrometry today presents one problem which calls for the use of a database to represent the information gained in this type of experiment. Since multiple databases are usually queried for the existence of an amino acid sequence that best explains an MS/MS spectrum, possibly using multiple software tools, the first task that needs to be achieved is the assurance that each mass spectrum is only represented by its best identification while making sure that it is also significantly better than the second best identification, if any. Furthermore, it is necessary to evaluate peptides based on the scores from different software tools; see Shadforth 2005 and Kapp 2005 for a review of the vast amount of available software [13,14]. This was achieved by allowing user defined thresholds for any potential software applications which can evaluate tandem mass spectra.

Peptides identified by distinct mass spectrometric identification algorithms have to be matched to possible amino acid sequences in order to discover putative proteins. In order to allow this assessment protein sequences need to be stored in the database. Any protein sequence available in FASTA format [15] can be imported into 2DB, which then builds the basis for automatic protein discovery from peptides. Interconnection to other databases, as stipulated for federated databases in an article involving SWISS-2DPAGE [16], has been adhered to in the implementation presented here. Our implementation does not aim to compete with SWISS-2DPAGE, rather, we aim to provide a solution which can be used to display all lines of evidence which led to protein identification, enabling the user to evaluate the result quality. Protein identification can, then, in the spirit of a federated database, be made available on the web and linked to, as well as searched from, SWISS-2DPAGE in the future.

## Implementation

There are currently two approaches that would allow for efficient representation of the wealth of information from proteomics experiments. One is to represent the data in extensible markup language (XML) [17] format and then use native XML database tools to access the information. It is questionable how this information, which is probably extremely heterogeneous, can be evaluated without extensive standardization approaches. Furthermore, there seems to be a need to rerun all MS/MS mapping algorithms and rebuild the database if the underlying protein models change. Another approach, used in this study, is to model the expected data to a relational database. This was done using a mixture of strict and loose typing in order to allow for the import of data from various sources, especially in regards to the results from computational identification of MS/MS spectra.

The database schema is currently specifically designed for the MySQL [18] database management system (DBMS) since it is powerful and free of charge. Furthermore, most web space providers support MySQL even in their basic packages. However, throughout the code plain structured query language (SQL) [19] was used which enables a smooth migration to other or multiple DBMS in the future. Most code has been written in hypertext preprocessor (PHP) [20] which is more widespread than MySQL and available on virtually any server on the internet. Three core applets were programmed in JAVA [21], a programming language executable on almost any computer having a recent version of the JAVA virtual machine installed. The interactive web pages have been designed in hypertext markup language (HTML) [22], JavaScript, and cascading style sheets (CSS) [23], The overall package should, therefore, work on most servers that are commercially available and on those provided in educational institutions.

The application is divided in four logical areas: administration, input, access and output. The database is accessed in a three tier mode. HTML forms build the front-end while PHP scripts provide the middleware which retrieves the information from the database and then displays the result to the user. Access control is based on users, groups, memberships to the groups, and rights. Login information is stored in encrypted cookies, which provides an adequate degree of security considering the sensitivity of the stored data.

The package can be downloaded as a single compressed file. After decompression and uploading to the server, a wizard style installation script will ensure that the application will be properly installed on the server. Thereafter, some customization is still necessary, such as importing possible amino acid sequences, entering users, and adding at least one organism before the application can work properly with imported data sets.

**Figure 1:**
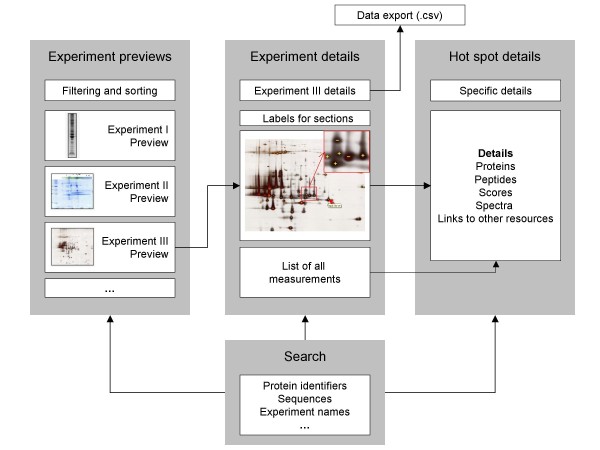
**Graphical user interface overview**. The user interface provides multiple ways to access information stored in the database. The experiment preview section shows previews of experiments associated with an image in the database. These previews are linked to details which include more elaborate descriptions of the experiments, and, if available, a clickable image map for each experiment with indication whether confident protein identification has been achieved for that area. A list of all measured spots is available, regardless of whether an image is provided. Hot spots or list of spots lead to the detailed protein, peptide, and MS information stored in the database. This section shows confident protein identifications at the top, with all other peptide identifications listed below. All areas can be reached via the search facility provided in the user interface. A summary of information regarding the confidently identified proteins can be exported as a text file with comma-separated values, which can be imported by most spreadsheet applications.

Figure 1 shows a brief overview of the user interface which provides access to the information stored in the database. Since it is the intention to give a general overview, no screen shots are shown, but the content is summarized. The implementation can be reviewed *in situ *on an example instantiation of the project (see below). Other connections between different user interaction screens to modify data stored in the database can be presented in a similar fashion.

## Results and discussion

Many databases that host proteomics data have been proposed, most of which are tailored to a specific purpose, organism, lab, or more generally, an environment [24-26]. This is also partly true for our implementation. It is directed towards proteomics experiments including single or multiple separation steps, followed by mass spectrometry and computational analysis of the MS/MS data. This includes, for example, 1D gels, 2D gels, and multidimensional protein identification technology (MudPIT) [27]. Within this context, however, we aim to represent all necessary lines of evidence leading to distinct protein identifications.

The 2DB application consists of four distinct areas which will be discussed in the following individual sections.

### Installation and administration

The first criterion for any software is that it needs to provide an easy-to-use installation procedure. Most current applications, however, expect the user to manually edit scripts, thus creating a large obstacle seriously limiting the software's potential for use. We overcame this by completely hiding this process from the user, only providing a wizard like interface to collect the necessary data for the installation of 2DB on a server.

Following installation, the administration section of 2DB should be the first stop. Usually a number of groups and users modelling differential access to the data should be created, which can be achieved in the groups and user-profile areas of the administration area of the user interface of 2DB (see Access Control below and additional file 2). The next important step is to enter the thresholds for the software tools used for peptide identification from MS/MS spectra which will provide the input to any instance of 2DB. This enables the database application to filter input data according to these thresholds.

After successful installation some initial customization is required for the efficient use of the database application. This includes the import of amino acid sequences (e.g. gene models) specific to the research organism.

### Import and data description

Any database application in proteomics should allow for batch import of data. However, other database applications, such as SWISS-2DPAGE [28] and PHProteomicDB [29], however, expect manual data entry for modelling protein identifications. Even though the amount of data needing to be entered is significantly lower than the amount of data which would need to be entered into 2DB, it is still inconvenient. Therefore, we provide complete automatic import of peptide identifications. Currently, we support the import of MS/MS identifications from Sequest [7] out, html, and xml files. As well as from X!Tandem [30] and Omssa [31] in csv and xml format. PepXML [32] files can also be imported in addition to AutoMS ams files (Allmer, not published). These formats can automatically be converted for upload to 2DB, using a special data format described in the manual (see Additional file 2). This format can also be used to import data from various other sources not yet specifically supported. It consists of only 20 data points in plain text format, some of which may be left empty. Adhering to this format ensures that the correct data items are grouped and related automatically.

In order to enable automatic protein discovery from this data, potential protein sequences have to be available in the database.

As mentioned earlier, the results are first filtered by user thresholds stored in the database. If the thresholds are not passed, the results are discarded. If the thresholds are passed, the result is stored in the database. Because different algorithms may perform the analysis and different sequence databases may be used, a mass spectrum can be mapped to different possible peptide sequences. This would lead to the undesirable effect of multiple sequences for a single MS/MS spectrum, and can be avoided by using cosine similarity to determine which sequence prediction is closer to the experimental spectrum. This assessment, while seeming crude, is deemed to be sufficient since results have been pre-filtered by other algorithms. In cases where the scores are identical, both results are discarded; if one scores better than the other, the lower score is discarded. We chose to keep the database free of the results that fail to pass the thresholds or that conflict, since the data inflation caused by this may lead to slow response times of the database management system as was experienced in preliminary trials.

We created a wizard-like interface able to import sequences in FASTA format. If the header (also known as identifier, or description line) information in the FASTA file contains protein identifiers that can be used to link to protein details, this information can be used to establish a link from the sequence in the database to the origin of the protein (e.g.: NCBI [33]). This is also possible if the underlying sequences come from multiple sources.

Each peptide imported after computational analysis of mass spectrometric experiments is automatically compared to the sequences stored in the database. If a match can be established, a link is formed in the database. Subsequently, a check is made to see whether, according to the confidence thresholds for protein identification, proteins can be identified from the relationships between the number of peptides identified in respect to a specific protein.

Many current databases fail to respond to changes in the underlying protein models since they are built on static models such as XML or HTML files. In the case of 2DB, the peptide protein link is established whenever changes in either the protein models or the stored identifications are made.

**Figure 2:**
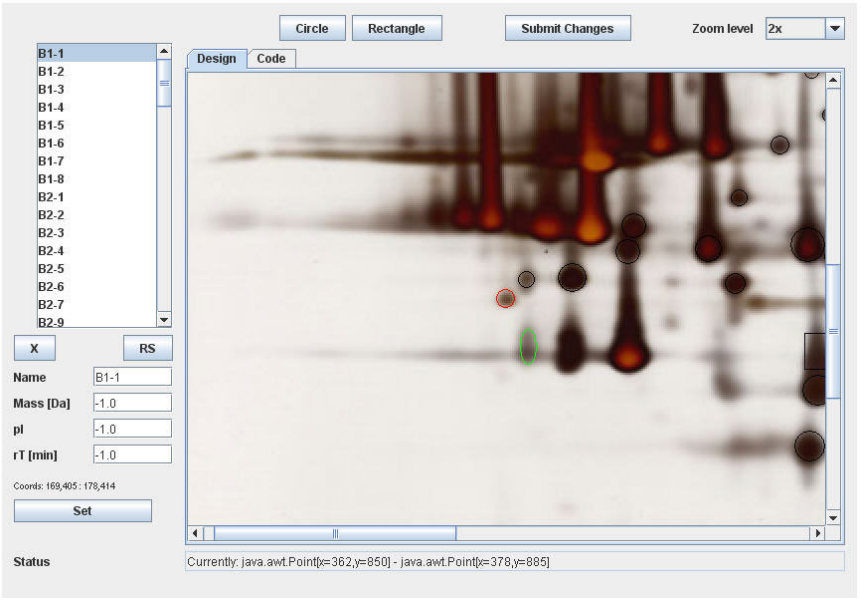
**Creating a clickable image map**. This JAVA applet, when called from the 2DB environment, displays the image of the experiment and the list of items that led to identifications. This list can now be correlated to the picture by clicking an item in the list and then defining an area on the picture. Additional information, such as mass and pI for the specific area, can be entered. At any point, the changes can be uploaded to the database and will persist there. Defining these hotspots essentially creates a clickable image map which effectively visualizes the data in the database.

The data imported into the database can be complemented with visual information; it is possible to upload a picture of a gel, blot, or similar item and correlate it to the imported data via hot spots on the image. These hotspots can be defined using a JAVA applet included in the 2DB package (Fig. 2). In addition to this, information concerning the experiment, such as methods employed and the overall aim of the study, can be entered. While considering this approach sufficient, we recognize the importance of standards, and intend to integrate support for HUPO's PSI-Gel [34] standard, which will describe, for example, spots on a gel, upon publication.

One of the most important databases representing results from proteomics experiments is SWISS-2DPAGE. It represents the identified proteins which must be established prior to constructing the 2D reference map. In our application proteins are only presented by sparse information and a hyperlink to their origin, whereas SWISS-2DPAGE gives additional information for each protein. While SWISS-2DPAGE only links MS/MS datasheets statically, our implementation actively uses MS/MS search results to discover proteins (see Automatic Protein Detection), which can subsequently be connected to areas on an image resource using a tool provided with the database (Fig. 2). Our database can be accessed in the spirit of a federated database, as described in a publication involving SWISS-2DPAGE [16]. This means that proteins can be searched for and their location identified as well (from an external link, if necessary) as it is possible to directly link to any publicly available section of this package.

Although data generated by proteomics experiments is vast and heterogeneous, and calls for a distributed representation, there is at least one approach which attempts to capture a large fraction of the information: This approach, called PEDRoDB [35], is based on data contained in XML files. This data can be queried with native XML database management systems such as XIndice [36] which was used in the above approach. Generally large data inflation is caused by capturing all available information, such as the parameters of the mass spectrometer over time, even more so if it involves XML which amends each data point with a data description (tag), sometimes more than doubling the size of the actual data. In certain environments it would be crucial to retain this information; however, for high throughput proteomics studies, some of this information may not be required in the context of peptide and protein identification. As a side effect, the additional information needs to be entered into the database by the experimenter. In order to reduce the workload on researchers as much as possible, we decided to take a lean approach representing all lines of evidence for each protein identification in a consistent manner while retaining only sparse evidence in raw format, such as mass spectra, which are an important means for assessing the identification quality.

PEDRo, intended as a model for capturing all data in gel-based proteomics, has been adopted by proteomics applications, for example MASPECTRAS, and forms the basis of its data model [37], which is similar to other laboratory information systems [38]. Recently the proteomics standard initiative formalized the minimum information necessary for a proteomics experiment [39] which now supersedes the PEDRo model.

2DB spans several parts of the MIAPE standard such as the gel electrophoresis, the mass spectrometry and the capillary electrophoresis modules.

In the 2DB database model, we tried to model information as abstract as possible so that even unexpected data can be captured: The scores provided for the peptide assignment to an MS/MS spectrum, for example, can be stored irrespective of the software used for the analysis and irrespective of their type or range.

We acknowledge the need for standardization and we are working towards compliance. At this point, 2DB is mainly designed to discover information from a subset of the captured data, but it also provides functionality to capture a large amount of meta data which can be tailored to compliance with standards upon their publication.

### Automatic protein detection

Peptides that are confidently identified from mass spectrometric data using established algorithms are related to protein sequences present in the database. Currently, it is possible to specify thresholds for the number of peptides that support a protein found in a given selection. This, for example, enables distinct views of the results with different levels of confidence. It is also possible to put more weight on peptides which have been identified by trusted applications. Combinations of both thresholds are also supported. In the future, we will add support for discrimination using the number of supporting spectra per identified peptide and the number of algorithms supporting the identification per spectrum.

A fraction which will be analyzed for identifiable proteins is currently set to be a subset of the experiment (e.g. spot/band on a gel). Peptides found in that fraction are pooled and mapped to the sequences in the database. The pooling of specific fractions or experiments is still at an experimental stage, but will be supported in the future. Thus, the results of multiple biological samples can then be pooled.

The analysis is done on the fly, so by adding new data to the database the confidently identified proteins are updated if affected by the addition.

### Access control and search facility

One aim of the software developed in this study is to allow concurrent storage, analysis, and publication of data bundled in one application. Therefore, a strict user control, achieved by introducing group level access rights, is essential. Thus it is possible to limit the access to data to any number of groups defined in the group section of the database. Two groups, administrators and guests, are initially set-up, although other groups can be added by administrators at any time. We currently have four groups, administrators, researchers, collaborators, and guests. Administrators can change and modify all parts of the database, while on the next level, the researchers from the host lab are only able to enter and retrieve data. Collaborators from other research groups are on the next lower level of access rights; they are only able to access data provided from the host specifically to them. Lastly, guest users can access all information which has been published, and is therefore in the public domain.

Given that the information sought for is actually accessible, the question remains as to how to retrieve information to which a user is entitled, according to the access rights previously described. We provide a basic search facility which can aid in finding information. Specific questions that can be asked are, for example, protein ids which are dependent on the sequences present in the database. We use transcript numbers provided by the Joint Genome Institute [40] as protein identifiers in the case of our working instance of the database, namely WWUPepProtDB[41].

The database has the advantage that, when a particular protein is searched for, its location is revealed in every experiment in which it was detected. This offers the possibility of finding differential expression of a protein in related experiments. Other search modes include sequence search, which not only retrieves proteins but also peptides, and searching for names of experiments or organisms. These are performed as a free text search which returns results from all areas of interest.

Other databases such as ProDB [42] and UAB [43] also provide the possibility for browsing locally produced data in a similar way to 2DB, although, there is a separation between the data presented to the research community and the data which is used in-house for data evaluation. 2DB offers the possibility of hosting both, the sensitive in-house information and the published data on the same server. It is sufficient to change the access rights in one location in order to open or close public access to an experiment in the database.

### Data views and output formats

**Figure 3:**
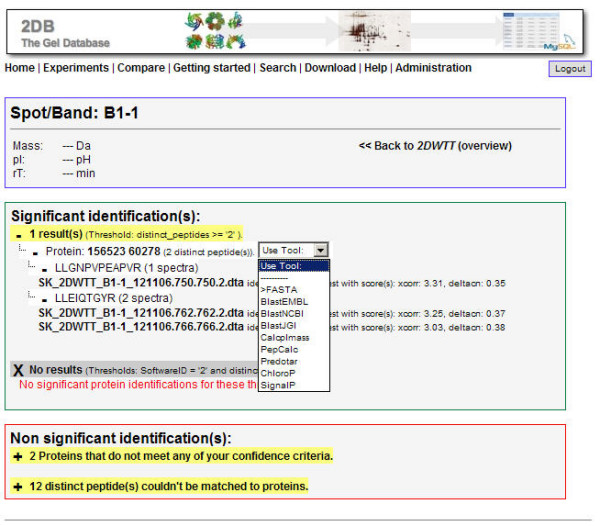
**Protein view and lines of evidence**. The spot details page provides information about peptide and protein identification from, for example, one spot on a 2D gel. First, the name and additional information is presented (here B1-1 see top box). Those proteins which pass the significance criteria set for protein identification from peptide identifications are presented in the second box. Multiple thresholds can be used to analyze the data more elaborately. Here only the first of two possible thresholds yields confident protein identifications. One protein with two distinct access numbers (for the same sequence; 156523 and 60278) was successfully identified by two supporting peptides with one and two MS/MS spectra, respectively. Both protein identifiers and spectrum ids are hyperlinks that lead to more detailed information. The drop-down box displays the plug-ins which are available to analyze the underlying sequence using different tools. The final box shows potentially interesting results, which have not lead to confident protein identifications when applying the thresholds. Neither result is expanded, although details are accessible by clicking the plus sign in front of either section. The first section hosts peptides that map onto amino acid sequences in the database whereas the second shows all those peptides that could not be matched.

The main entry points for accessing information on the 2DB platform are the search facility and the experiment gallery sections. All experiments stored in the database can be accessed via the experiment gallery. Access rights are indicated by a green box (sufficient) or a red frame (insufficient). Clicking on one of the previews, if available, brings up the experiments details page which includes all information about the experiment as provided by the experimenter and a larger view of the image representing the experiment, if available. This picture can serve as an image map and can lead to more detailed information about proteins identified for areas of the image (Fig. 3). Following such a link will display all proteins on top of the result page that were confidently identified from the recorded mass spectra for that spot. This is followed by proteins which lack enough supporting peptides, and thus do not pass the significance thresholds. Finally, peptides which cannot be mapped to protein sequences in the database are listed. The information is presented in such a way that details need to be specifically accessed. On the first level, the identified proteins are displayed. Enabling the second layer will provide all supporting peptides for a particular protein. Each supporting peptide may be identified by a single, or multiple spectra which can be seen on the following level. Each supporting spectrum can be viewed in context with the theoretical fragmentation pattern of the predicted amino acid sequence. Furthermore, each spectrum, which led to the same proposed peptide, comes with the scores given by each software tool used for its identification and Meta-data (in other words: data about data) can be generated and downloaded as a table. An extract of an experiment is given as an example (Tab. 1) which can be downloaded from the experiment overview. This table contains information about the protein identifications from an experiment, such as the number of spectra and peptides required for confident protein identification.

**Table 1:** Data export format.

**Band|Spot**	**Protein**	**Description**	**Spectral Count**	**Distinct Peptides**	**Sequence Coverage**	**Sequest (normal/intron split)**	**GPF-Sequest (normal/intron split)**
B1-1	156523 60278	estExt_GenewiseH_1.C_820026	5	2	0.08	2|-	-|-
B1-5	153056	153056	15	4	0.237	4|-	2|-
B1-5	78552	estExt_GenewiseW_1.C_140139	15	4	0.237	4|-	2|-
B1-6	153056	153056	14	4	0.207	4|-	2|-
B1-6	78552	estExt_GenewiseW_1.C_140139	14	4	0.207	4|-	2|-
B1-7	163297	163297	11	4	0.261	4|-	-|-
B1-7	24036	estExt_fgenesh1_pm.C_40007	11	4	0.28	4|-	-|-
B1-8	156858 132678	estExt_gwp_1H.C_660032	11	2	0.098	1|1	1|1
B2-2	153056	153056	23	5	0.324	5|-	2|-
B2-2	155433 133575	estExt_gwp_1W.C_80187	23	4	0.261	4|-	-|-
B2-2	78552	estExt_GenewiseW_1.C_140139	23	5	0.324	5|-	2|-
B2-3	158139	fgenesh2_kg.C_scaffold_74000005	5	1	0.035	1|-	1|-
B2-4	165269	165269	6	1	0.053	-|-	1|-
B2-5	158876 184810	estExt_fgenesh2_kg.C_320006	9	1	0.043	1|-	1|-
B2-6	155433 133575	estExt_gwp_1W.C_80187	22	3	0.21	3|-	-|-
B2-6	163297	163297	22	6	0.385	6|-	1|-
B2-6	24036	estExt_fgenesh1_pm.C_40007	22	6	0.413	6|-	1|-
B2-9	155433 133575	estExt_gwp_1W.C_80187	12	5	0.315	5|-	1|-

This table shows a section of meta-data about identifications we were able to make in an experiment. The exported data contains only meta-information about the identified proteins. This information includes the protein identifier, the number of supporting peptides, the sequence coverage and other information. Files with comma separated values (csv) can be imported in basically all spreadsheet calculation software such as Microsoft Excel, Systat's SigmaPlot and OpenOffice's Calc.

Other tools that are available but subject to on going development are, for example, the calculation of differential protein expression in different experiments stored in the database. This calculation is currently based on spectral and peptide count only.

Links to other databases are provided through the name of the protein or by plug-ins, which can be used to send the underlying protein sequence to different websites in order to retrieve further information. Plug-ins are extremely easy to define, although, the number presently available seems sufficient at this point. Other implementations such as ProteomeWeb [44] provide functionality, for example the calculation of theoretical maps, not currently implemented in 2DB due to lack of manpower.

There are more than 20 experiments in our local instance of the 2DB application [41] four of which are publicly accessible and which were recently published in Proteomics [11].

## Conclusion

Many databases have been tailored to specific purposes in the past. Here we present a database application which can be employed in many contexts, regardless of the software framework currently used. Although the database application developed in this study can represent identified proteins in their experimental context, much like SWISS-2DPAGE, it can also store selected mass spectrometric raw data to provide all necessary lines of evidence to back up each of the protein identifications. A mixture of loose and strict typing within a relational database context makes it possible to integrate data from multiple sources which means that virtually any software for identification of MS/MS spectra can be used. Furthermore, the move away from a static model to a more dynamic model fits changes in underlying protein models very well. In summary, the database greatly facilitates proteomic data handling as well as analysis from high throughput experiments.

## Outlook

While the database is fully functional, there are some aspects that can be improved upon as can be seen in one instance WWUPepProtDB [41]. For example, at present, contradicting information given by different algorithms is resolved via cosine similarity, whereas other algorithms may be more suitable for this purpose. This implementation is at present limited in regards to input formats which can be handled by the file upload software. We will, however, implement converters and import facilities for standards as they become available.

## Availability and requirements

• The project sources and installation script can be downloaded from our webpage [12]. Help is available on the Google group [45].

• Experiments serving as a sample preview in the context of this paper are presented at the web on WWUPepProtDB [41].

• Operating system: The project was developed on a UNIX server, but should generally not be sensitive to a specific operating system.

• Browser: The database is only accessed via a web browser. It has been tested with Internet Explorer 6 and 7, FireFox 2.0, Safari 3.0, and Opera 9.1. In Internet Explorer 7, privacy and security settings need to be adjusted to be able to log onto the system.

• Implementation details: PHP was used in combination with HTML, CSS, and JavaScript for most parts of the user interface. JAVA was used for extensive user interaction modules. The database was developed using SQL, but was only tested using the MySQL database management system.

• Requirements: Any web server able to host MySQL 4.1 or greater and PHP 4 or greater. We used several versions of Apache. JVM 1.5 or greater, preferably from Sun Microsystems needs to be installed on the client computers. JAVA, JavaScript, and cookies need to be enabled in the web browser.

• The server can be run locally on a PC as well. We achieved the best results using WampServer 2 [46].

• License: GNU General Public License

## Authors' contributions

JA designed the database schema and programmed significant parts of the scripts. He also wrote the first draft of this publication. SK designed the user interface and programmed significant parts of the scripts. He proofed all versions of this publication. MH supervised and provided constant critiques and insights throughout the development of the application. He significantly revised and enhanced the first draft of this publication.

## Supplementary Material

Additional file 1 All files needed to run and further develop the database application as well as the user manual have been bundled into one zip file which can be downloaded from biomedcentral here. Due to constant upgrading of the system, it may be beneficial to check for the latest version on our website [12]. All the sources and additional installation files. Click here for file

Additional file 2 All files needed to run and further develop the database application as well as the user manual have been bundled into one zip file which can be downloaded from biomedcentral here. Due to constant upgrading of the system, it may be beneficial to check for the latest version on our website [12]. The user manual for both installation and usage. Click here for file
