# Supplementary material for: 2DB: a Proteomics database for storage, analysis, presentation, and retrieval of information from mass spectrometric experiments
Source: BMC Bioinformatics. 2008 Jul 7;9:302. doi: 10.1186/1471-2105-9-302 (PMC2475538; doi:10.1186/1471-2105-9-302)
Supplement: Additional file 1 — All files needed to run and further develop the database application as well as the user manual have been bundled into one zip file which can be downloaded from biomedcentral here. Due to constant upgrading of the system, it may be beneficial to check for the latest version on our website [12]. All the sources and additional installation files. [file 1471-2105-9-302-S1.zip › compare.php]

2DB - Compare Experiments
php include("layout/menu.php"); ?

## Compare Experiments

This is a simple function to compare two experiment images.  
  
php
//////////////////////////////////////////////////
// A simple way to compare two images //
//////////////////////////////////////////////////
if (!isset ($\_COOKIE["login"])){
echo Warning("bad", "You have to be logged in to use this funktion!");
}
else{
echo "<form action=\"compare.php\" method=\"post\"\n";
echo "

\n";
echo "|  |  |
| --- | --- |
|\n";
echo " **Choose Experiments:** |\n";
echo "  |\n";
echo "
\n";
echo "|\n";
echo "  |\n";
echo "
\n";
echo "|\n";
echo " \n"; echo "\n"; $rs = GetResultTableSQL("SELECT ID, Name, Date, Image FROM Separations WHERE Image != ''"); if(!$rs){ echo"----------\n"; }else{ for($i=0; $i$row[1] ($datum)\n"; } } echo "\n"; echo " |\n";
echo " \n"; echo "\n"; if(!$rs){ echo"----------\n"; }else{ $rs = GetResultTableSQL("SELECT ID, Name, Date, Image FROM Separations WHERE Image != ''"); for($i=0; $i$row[1] ($datum)\n"; } } echo "\n"; echo " |\n";
echo "|\n";
echo "

  
\n";
echo "\n";
echo "\n";
echo "  
\n";
echo "  
\n";
if($compare == "compare"){
if(!$gel1 or !$gel2){
echo Warning("bad", "Sorry, but there are no images available!");
}else{
$path = "gelimages/pics/"; // Path for the images
$max\_width = 400; // maximum width
$max\_height = 400; // maximum width
$size\_1 = getimagesize ("$path$gel1");
$size\_2 = getimagesize ("$path$gel2");
$width\_1 = $size\_1[0];
$height\_1 = $size\_1[1];
$width\_2 = $size\_2[0];
$height\_2 = $size\_2[1];
$ratio\_1 = $max\_width / $width\_1;
$test\_1 = $height\_1 \* $ratio\_1;
if($test\_1 > $max\_height){
$ratio\_1 = $max\_height / $height\_1;
}
$n\_width\_1 = $width\_1 \* $ratio\_1;
$n\_height\_1 = $height\_1 \* $ratio\_1;
$ratio\_2 = $max\_width / $width\_2;
$test\_2 = $height\_2 \* $ratio\_2;
if($test\_2 > $max\_height){
$ratio\_2 = $max\_height / $height\_2;
}
$n\_width\_2 = $width\_2 \* $ratio\_2;
$n\_height\_2 = $height\_2 \* $ratio\_2;
echo "

\n";
echo "|  |  |
| --- | --- |
|\n";
echo " \n"; echo "\n"; echo " |\n";
echo " \n"; echo "\n"; echo " |\n";
echo "|\n";
echo "

\n";
echo "  
\n";
echo "  
\n";
}
}
}
?>
php include("layout/footer.php"); ?
