# Supplementary material for: 2DB: a Proteomics database for storage, analysis, presentation, and retrieval of information from mass spectrometric experiments
Source: BMC Bioinformatics. 2008 Jul 7;9:302. doi: 10.1186/1471-2105-9-302 (PMC2475538; doi:10.1186/1471-2105-9-302)
Supplement: Additional file 1 — All files needed to run and further develop the database application as well as the user manual have been bundled into one zip file which can be downloaded from biomedcentral here. Due to constant upgrading of the system, it may be beneficial to check for the latest version on our website [12]. All the sources and additional installation files. [file 1471-2105-9-302-S1.zip › contact.php]

2DB - Contact
php include("layout/menu.php");?

## Contact

php
$rs = GetResultTableSQL("SELECT Value FROM Misc WHERE Misc.Name='Contact Name'");
echo "<b" .$rs[0][0]. "  
";
$rs = GetResultTableSQL("SELECT Value FROM Misc WHERE Misc.Name='Contact Institute'");
echo $rs[0][0]. "  
";
$rs = GetResultTableSQL("SELECT Value FROM Misc WHERE Misc.Name='Contact Zip'");
echo $rs[0][0]. " ";
$rs = GetResultTableSQL("SELECT Value FROM Misc WHERE Misc.Name='Contact City'");
echo $rs[0][0]. "  
";
$rs = GetResultTableSQL("SELECT Value FROM Misc WHERE Misc.Name='Contact State'");
echo $rs[0][0]. "  
";
$rs = GetResultTableSQL("SELECT Value FROM Misc WHERE Misc.Name='Contact Country'");
echo $rs[0][0]. "  
";
$rs = GetResultTableSQL("SELECT Value FROM Misc WHERE Misc.Name='Contact Email'");
if(!$rs){
echo "";
}else{
echo "  
";
}
$rs = GetResultTableSQL("SELECT Value FROM Misc WHERE Misc.Name='Contact URL'");
echo "" .$rs[0][0]. "  
";
echo "  
";
include("layout/footer.php"); ?>
