# Supplementary material for: 2DB: a Proteomics database for storage, analysis, presentation, and retrieval of information from mass spectrometric experiments
Source: BMC Bioinformatics. 2008 Jul 7;9:302. doi: 10.1186/1471-2105-9-302 (PMC2475538; doi:10.1186/1471-2105-9-302)
Supplement: Additional file 1 — All files needed to run and further develop the database application as well as the user manual have been bundled into one zip file which can be downloaded from biomedcentral here. Due to constant upgrading of the system, it may be beneficial to check for the latest version on our website [12]. All the sources and additional installation files. [file 1471-2105-9-302-S1.zip › download.php]

2DB - Download
php include("layout/menu.php"); ?

## Download

You can only download the most current version of the 2DB database.  
While we are continuously developing, updates may become available  
which you can then incorporate using your instance of the database.

  
  
Please go to http://www.2db.de.ms or
http://www.biolnk.com/2db/ to find the download.
  
  
php
if (isset ($\_COOKIE["login"]))
{
//The server is hardcoded here. It may be useful to make it a database entry in Misc.
$h = @fopen("http://www.biolnk.com/2db/install/version.php","r");
if($h) {
while (!feof($h)) {
$remoteVersion .= fgets($h, 4096);
}
pclose($h);
if(strlen($remoteVersion)  2) {
settype($remoteVersion,"double");
$rs = GetResultTableSQL("SELECT Value FROM Misc WHERE name='version'");
if($rs)
$localVersion = $rs[0][0];
else
$localVersion = 0.8;
settype($version,"double");
if($localVersion < $remoteVersion) {
echo"A new version (2DB $remoteVersion) of the database schema or GUI enhancements are available.  
";
//echo"if you would like to upgrade please click the following link.  
  
";
//echo"Upgrade  
  
";
//echo"Make sure that you are logged-on as an administrator. Otherwise the script will fail.  
";
} else
echo"This instance of 2DB is the most current version.  
";
} else {
echo "Unexpected response from remote server  
";
}
} else
echo"Couldn't connect to upgrade service  
";
}
?>
php include("layout/footer.php"); ?
