# Supplementary material for: 2DB: a Proteomics database for storage, analysis, presentation, and retrieval of information from mass spectrometric experiments
Source: BMC Bioinformatics. 2008 Jul 7;9:302. doi: 10.1186/1471-2105-9-302 (PMC2475538; doi:10.1186/1471-2105-9-302)
Supplement: Additional file 1 — All files needed to run and further develop the database application as well as the user manual have been bundled into one zip file which can be downloaded from biomedcentral here. Due to constant upgrading of the system, it may be beneficial to check for the latest version on our website [12]. All the sources and additional installation files. [file 1471-2105-9-302-S1.zip › experiment_detail.php]

2DB - Experiment Details
php
include("layout/menu.php");
$gel = $id;
$uid = $tab[1];
//First check whether the user is eligible to access the data.
if(!CanAccess($gel,$uid)) {
?
php
}
///////////////////// Gel Details /////////////////////////////
$rs = GetResultTableSQL("SELECT \* FROM Separations WHERE ID ='$gel'");
$row\_detail = $rs[0];
echo "<h2 class=\"title\"Experiment: $row\_detail[1]\n";
echo "

";
$pub = GetResultTableSQL("SELECT Link,Display,p.ID FROM Publications AS p INNER JOIN Links AS l ON l.PublicationID = p.ID WHERE p.SeparationID='$gel'");
echo "

";
if($pub) {
echo "|  |  |
| --- | --- |
|";
echo " **Published in:** |";
echo " "; for($p=0; $p". $pub[$p][1] . " "; } echo " |
";
}
echo "|";
echo " **Separation Method:** |";
echo " $row\_detail[2] |";
echo "
";
echo "|";
echo " **Organism:** |";
/////////////// Organism //////////////////
$rs = GetResultTableSQL("SELECT Genus, Species, Strain FROM Organisms WHERE ID = '$row\_detail[4]'");
$row\_organism = $rs[0];
///////////// Organism End ///////////////
echo " *$row\_organism[0] $row\_organism[1]* - $row\_organism[2] |\n";
echo "
\n";
echo "|\n";
echo " **Detail Information:** |\n";
echo " $row\_detail[9] |\n";
echo "
\n";
echo "|\n";
echo " **Significant Results:** |\n";
echo " [show] - [download] |\n";
echo "
\n";
echo "

\n";
echo "

\n";
echo "  
\n";
////////////////////// Visual help ///////////////////////
if($row\_detail[6] != ""){
echo "

";
echo "\n";
echo "

";
echo "|  |  |
| --- | --- |
|\n";
echo " "; echo "**Visual help for finding identified spots** (lower image quality): \n"; echo " |\n";
echo " "; echo "Marker color: \n"; if($color == "255,0,0" /\*or $color == ""\*/){$c1 = "checked=\"checked\"";} echo "      \n"; if($color == "0,255,0"){$c2 = "checked=\"checked\"";} echo "      \n"; if($color == "0,0,255"){$c3 = "checked=\"checked\"";} echo "      \n"; if($color == "255,255,0"){$c4 = "checked=\"checked\"";} echo "      \n"; if($color == ""){$c5 = "checked=\"checked\"";} echo "      \n"; if($color == ""){$c6 = "checked=\"checked\"";} echo "none\n"; echo ""; echo "  "; echo "Caption: + identified | - measured"; echo " |\n";
echo "
\n";
echo "

\n";
echo "\n";
echo "

\n";
echo "  
\n";
echo "\n";
////////////////// Writes Imagemap ///////////////////////
$rs = GetResultTableSQL("SELECT \* FROM Fractionations INNER JOIN Shapes ON Fractionations.ID = Shapes.FractionationID WHERE SeparationID='$gel' AND Shapes.Shape IS NOT NULL");
for($i=0; $i\n";
} else {
echo "\n";
}
}
//////////////////////////////////////////////////////////
echo "\n";
$image = "gelimages/pics/$row\_detail[6]";
$size = getimagesize( $image );
if($map == "help" and $color != ""){
$imagesrc = "gelimages/picture.php?gelid=$id&color=$color";
}
else{
$imagesrc = $image;
}
echo "\n";
echo "  
\n";
echo "  
\n";
}
//////////////////////// Links to all measured spots//////////////////////////////////
echo "

\n";
echo "**All measured Spots:**  
\n";
echo "

\n";
echo "|  |  |
| --- | --- |
|\n";
$rs = GetResultTableSQL("SELECT Fractionations.ID, Fractionations.Name FROM Fractionations WHERE SeparationID='$gel' ORDER BY Fractionations.Name");
for($i=0; $i\n|  |  |
| --- | --- |
|\n";
$j=0;
}
echo " $spotName |\n";
$j++;
}
for($k=$j; $k<10; $k++) {
echo "  |\n";
}
echo "
\n";
echo "

\n";
echo "

\n  
\n";
?>
php include("layout/footer.php"); ?
