# Supplementary material for: 2DB: a Proteomics database for storage, analysis, presentation, and retrieval of information from mass spectrometric experiments
Source: BMC Bioinformatics. 2008 Jul 7;9:302. doi: 10.1186/1471-2105-9-302 (PMC2475538; doi:10.1186/1471-2105-9-302)
Supplement: Additional file 1 — All files needed to run and further develop the database application as well as the user manual have been bundled into one zip file which can be downloaded from biomedcentral here. Due to constant upgrading of the system, it may be beneficial to check for the latest version on our website [12]. All the sources and additional installation files. [file 1471-2105-9-302-S1.zip › experiment_gallery.php]

Experiment Gallery
php
include("layout/menu.php");
////////////////////////////////////////////////////
//// ////
//// Experiment gallery with access controll ////
//// ////
////////////////////////////////////////////////////
if(!$tab[0]){
$rs = GetResultTableSQL("SELECT a.RowID, a.GroupID, g.Name FROM Access as a INNER JOIN Groups as g ON a.GroupID=g.ID WHERE g.Name = 'Guests'");
for($i=0; $i<count($rs); $i++) {
$group = $rs[$i];
$a = $group[0];
$grp\_access[$a] = $group[0];
}
$access=1;
}
if($tab[0] == "true"){
$rs = GetResultTableSQL("SELECT Name FROM Members INNER JOIN Groups ON Members.GroupID = Groups.ID WHERE UserID='$tab[1]' AND Name = 'Administrators'");
if(!$rs){
$rs = GetResultTableSQL("SELECT a.RowID, a.GroupID, m.UserID, g.Name FROM Access as a INNER JOIN Members as m ON a.GroupID=m.GroupID INNER JOIN Groups as g ON m.GroupID=g.ID WHERE m.UserID='$tab[1]'");
for($i=0; $i<count($rs); $i++) {
$group = $rs[$i];
$a = $group[0];
$grp\_access[$a] = $group[0];
}
$rs = GetResultTableSQL("SELECT a.RowID, a.GroupID, g.Name FROM Access as a INNER JOIN Groups as g ON a.GroupID=g.ID WHERE g.Name = 'Guests'");
for($i=0; $i<count($rs); $i++) {
$group = $rs[$i];
$a = $group[0];
$grp\_access[$a] = $group[0];
}
$access=1;
}else{
$full\_access=1;
}
}
//////////////////////// Navigation ////////////////////////
if(!$start)
$start = 0; ////// Sets the Startpoint for the first time to 0
if(!$show)
$show = 10; ////// Set the Records for the first time to 10
if(!$sort)
$sort = "Date DESC"; ////// Set the Order for the first time to date desc
$rs = GetResultTableSQL("SELECT COUNT(ID) FROM Separations");
$sum = $rs[0];
$previous = $start-$show;
if($previous < 0)
$previous = -1;
$next = $start+$show;
if($next = $sum[0])
$next = $sum[0];
///////////////////////////////////// Gallery //////////////////////////////////////
echo "

## Experiments

\n";
echo "\n";
echo "

\n\n";
echo " Access:          No access: |\n";
echo " \n"; if($previous == -1) echo " "; else echo "« previous \n"; echo " |\n";
echo " \n"; if($next < $sum[0]) echo " next »"; else echo " "; echo " |\n";
echo " Display: |\n";
echo " "; echo ""; echo "1\n"; echo "5\n"; echo "10\n"; echo "25\n"; echo "50\n"; echo "\n"; echo " |\n";
echo " Filter: |\n";
echo " "; echo " "; echo " |\n";
echo " "; echo ""; echo "Sort by\n"; echo "Date\n"; echo "Method\n"; echo "Name\n"; echo "Organism\n"; echo "My Data\n"; echo "\n"; echo " |\n";
echo "  |\n\n";
echo "

\n";
echo "\n";
echo "  
";
if($sort == "OperatorID"){
$User = "Where OperatorID = '". $tab[1] ."'";
}else{
$User = "";
}
//Use the filter "author:name name" or "author:name,name"
// journal:name
if(isset($filter) && $filter != "") {
list($field,$constraint) = split(":",$filter,2);
mysql\_query("CREATE TEMPORARY TABLE ids SELECT ID FROM Users WHERE ID='-1'");
switch($field) {
case "author" :
if(strpos($constraint,",") !== -1) {
$cons = split(",",$constraint);
for($i=0; $i";
echo "

";
echo "|  |  |
| --- | --- |
|";
echo " "; if(($access == "1" and $row\_gallery[0] == "$grp\_access[$a]") || $full\_access == "1"){ echo ""; } if($row\_gallery[4] != ""){ echo " |";
}else{
echo "";
}
echo " "; echo "**Name:** $row\_gallery[1] "; // Get the Separation Method Name $sepNameQuery = GetResultTableSQL("Select Vocable FROM CV WHERE ID = '$row\_gallery[2]' Limit 1"); $sepName = $sepNameQuery[0]; echo "**Separation Method:** $sepName[0] "; /////////////// Organism ////////////////// $organism = GetResultTableSQL("SELECT Genus, Species, Strain FROM Organisms WHERE ID = '$row\_gallery[3]'"); $row\_organism = $organism[0]; $pub = GetResultTableSQL("SELECT Link,Display,p.ID FROM Publications AS p INNER JOIN Links AS l ON l.PublicationID = p.ID WHERE p.SeparationID='$row\_gallery[0]'"); ///////////// Organism End /////////////// echo "**Organism:** *$row\_organism[0] $row\_organism[1]* - $row\_organism[2] "; echo "**Description:** $row\_gallery[5]"; echo " |";
echo "
";
echo "|  | "; for($p=0; $p". $pub[$p][1] . " "; } echo " |\n
\n

\n";
echo "  
";
}
}
echo "

\n\n";
echo "  |\n";
echo " \n"; if($previous == -1) echo " "; else echo "« previous \n"; echo " |\n";
echo " \n"; if($next < $sum[0]) echo " next »"; else echo " "; echo " |\n";
echo "  |\n";
echo "

";
?>
php include("layout/footer.php"); ?
