# Supplementary material for: 2DB: a Proteomics database for storage, analysis, presentation, and retrieval of information from mass spectrometric experiments
Source: BMC Bioinformatics. 2008 Jul 7;9:302. doi: 10.1186/1471-2105-9-302 (PMC2475538; doi:10.1186/1471-2105-9-302)
Supplement: Additional file 1 — All files needed to run and further develop the database application as well as the user manual have been bundled into one zip file which can be downloaded from biomedcentral here. Due to constant upgrading of the system, it may be beneficial to check for the latest version on our website [12]. All the sources and additional installation files. [file 1471-2105-9-302-S1.zip › forget.php]

2DB - Forgot Password?
php include("layout/menu.php"); ?
**Forgot your password?**  
  
If you forgot your password please type in your email adress.  
A new password will be set and send to your email.  
You can change yor password in the Administration section under User-Profile.  
  


  
php
if($forget == "OK"){
$rs = GetResultTableSQL("SELECT ID FROM Users WHERE Email='$query'");
if(!$rs){
echo Warning("bad", "Your email coudn't be found. Please try again!");
}
else{
$row = $rs[0];
$Password = date("HdBy");
$coded = sha1($Password);
$rs = mysql\_query("UPDATE Users Set Password='$coded' WHERE ID='$row[0]'");
if(!$rs){
echo Warning("bad", "Coundn't write new password into DB!");
}
/////////// Send mail with login ///////
$eol="\r\n";
$header .= 'From: do\_not\_reply@2db.de.ms'.$eol;
$header .= 'Reply-To: do\_not\_reply@2db.de.ms'.$eol;
$header .= 'CC:'.$eol;
$header .= 'BCC:'.$eol;
$header .= 'X-Mailer: PHP/' . phpversion().$eol;
$betreff = "Your new $setname password";
$message = "Hello, \n \n here is your new password: $Password.\n\n If you are logged in you can change your password under Administration - User-Profile. ";
mail($query, $betreff, $message, $header);
////////////////////////////////////////
echo Warning("good", "Check your emails for your new password!");
}
}
?>
php include("layout/footer.php"); ?
