# Supplementary material for: 2DB: a Proteomics database for storage, analysis, presentation, and retrieval of information from mass spectrometric experiments
Source: BMC Bioinformatics. 2008 Jul 7;9:302. doi: 10.1186/1471-2105-9-302 (PMC2475538; doi:10.1186/1471-2105-9-302)
Supplement: Additional file 1 — All files needed to run and further develop the database application as well as the user manual have been bundled into one zip file which can be downloaded from biomedcentral here. Due to constant upgrading of the system, it may be beneficial to check for the latest version on our website [12]. All the sources and additional installation files. [file 1471-2105-9-302-S1.zip › index.php]

2D-Gel Database - Welcome to 2DB
php include("layout/menu.php"); ?

## php echo "$setname"; ?

| **Welcome** | |
| **Your Status:**  php if($tab[0] == "true") echo "Member - $tab[2] $tab[3]"; else echo "Guest"; ? | php if($tab[0] == "true"){ ? **Administration** Make Settings for php echo "$setname" ? php } else{ echo "&nbsp;"; } ? |
| **Experiments** | |
| **Experiments** Choose an Experiment | **Search** Search the Database |
| **Quantification** Quantify by Spectral Count | **Compare** Compare two Experiment Images |
| **About the Database** | |
| php $filename = "introduction.txt"; $handle = fopen($filename, "r"); $content = fread($handle, filesize($filename)); echo nl2br($content); fclose($handle); ? | |

  
php include("layout/footer.php"); ?
