# Supplementary material for: 2DB: a Proteomics database for storage, analysis, presentation, and retrieval of information from mass spectrometric experiments
Source: BMC Bioinformatics. 2008 Jul 7;9:302. doi: 10.1186/1471-2105-9-302 (PMC2475538; doi:10.1186/1471-2105-9-302)
Supplement: Additional file 1 — All files needed to run and further develop the database application as well as the user manual have been bundled into one zip file which can be downloaded from biomedcentral here. Due to constant upgrading of the system, it may be beneficial to check for the latest version on our website [12]. All the sources and additional installation files. [file 1471-2105-9-302-S1.zip › quantHTMLoutput.php]

2DB - Compare Experiments
php
include("layout/menu.php");
$sql = "Select Separations.Name from Separations Where Separations.ID = '$sep' Limit 1";
$rs = GetResultTableSQL($sql);
$experimentname = $rs[0];
?

## Quantification by Spectral Count for php echo "$experimentname[0]"; ? and

php
//////////////////////////////////////////////////
// Show the result list as html //
//////////////////////////////////////////////////
if (!isset ($\_COOKIE["login"])){
echo "<div style=\"padding:4px; border-color:#FF0000; border-width:1px; border-style:solid;\"**!** You have to be logged in to use this funktion!  
\n";
}
else{
?>

### Proteins found in both Experiments

Experiment 1:    
Experiment 2:   
  

php
echo "<tr";
echo " **Protein** - Description |";
echo " **Exp. - Band** |";
echo " **Distinct Peptides** |";
echo " **Distinct Spectra** |";
echo " **Method** |";
echo "";
$i=1;
$arr = file("test.csv");
foreach ($arr as $line) {
$count = $i;
list($protein,$aliasse,$description,$bandI,$distinct\_pepI,$distinct\_seqI,$bandII,$distinct\_pepII,$distinct\_seqII,$method) = split(',', $line);
if($bandI != "" and $bandII != ""){
if($i == 1){
echo "|";
echo " **$protein** $description |";
echo " "; if($bandI == ""){} else{ echo"$bandI";} if($bandI != "" && $bandII != ""){echo " ";} if($bandII == ""){} else{ echo"$bandII";} echo" |";
echo " "; if($bandI == ""){} else{ echo" $distinct\_pepI";} if($bandI != "" && $bandII != ""){echo " ";} if($bandII ==""){} else{echo " $distinct\_pepII";} echo " |";
echo " "; if($bandI == ""){} else{ echo" $distinct\_seqI";} if($bandI != "" && $bandII != ""){echo " ";} if($bandII ==""){} else{echo " $distinct\_seqII";} echo " |";
echo " $method |";
echo "
";
}else{
echo "|";
echo " "; if($bandI == ""){} else{ echo"$bandI";} if($bandI != "" && $bandII != ""){echo " ";} if($bandII == ""){} else{ echo"$bandII";} echo" |";
echo " "; if($bandI == ""){} else{ echo" $distinct\_pepI";} if($bandI != "" && $bandII != ""){echo " ";} if($bandII ==""){} else{echo " $distinct\_pepII";} echo " |";
echo " "; if($bandI == ""){} else{ echo" $distinct\_seqI";} if($bandI != "" && $bandII != ""){echo " ";} if($bandII ==""){} else{echo " $distinct\_seqII";} echo " |";
echo " $method |";
echo "
";
if($i == 4){
echo "|  |
";
$i=0;
}
}
$i++;
}
}
echo "

";
echo "

### Proteins found in Experiment 1

";
echo "

";
echo "|  |  |  |  |  |
| --- | --- | --- | --- | --- |
|";
echo " **Protein** - Description |";
echo " **Exp. - Band** |";
echo " **Distinct Peptides** |";
echo " **Distinct Spectra** |";
echo " **Method** |";
echo "
";
$i=1;
$arr = file("test.csv");
foreach ($arr as $line) {
$count = $i;
list($protein,$aliasse,$description,$bandI,$distinct\_pepI,$distinct\_seqI,$bandII,$distinct\_pepII,$distinct\_seqII,$method) = split(',', $line);
if($bandI != "" && $bandII == ""){
if($i == 1){
echo "|";
echo " **$protein** $description |";
echo " "; if($bandI == ""){} else{ echo"$bandI";} if($bandI != "" && $bandII != ""){echo " ";} if($bandII == ""){} else{ echo"$bandII";} echo" |";
echo " "; if($bandI == ""){} else{ echo" $distinct\_pepI";} if($bandI != "" && $bandII != ""){echo " ";} if($bandII ==""){} else{echo " $distinct\_pepII";} echo " |";
echo " "; if($bandI == ""){} else{ echo" $distinct\_seqI";} if($bandI != "" && $bandII != ""){echo " ";} if($bandII ==""){} else{echo " $distinct\_seqII";} echo " |";
echo " $method |";
echo "
";
}else{
echo "|";
echo " "; if($bandI == ""){} else{ echo"$bandI";} if($bandI != "" && $bandII != ""){echo " ";} if($bandII == ""){} else{ echo"$bandII";} echo" |";
echo " "; if($bandI == ""){} else{ echo" $distinct\_pepI";} if($bandI != "" && $bandII != ""){echo " ";} if($bandII ==""){} else{echo " $distinct\_pepII";} echo " |";
echo " "; if($bandI == ""){} else{ echo" $distinct\_seqI";} if($bandI != "" && $bandII != ""){echo " ";} if($bandII ==""){} else{echo " $distinct\_seqII";} echo " |";
echo " $method |";
echo "
";
if($i == 4){
echo "|  |
";
$i=0;
}
}
$i++;
}
}
echo "

";
echo "

### Proteins found in Experiment 2

";
echo "

";
echo "|  |  |  |  |  |
| --- | --- | --- | --- | --- |
|";
echo " **Protein** - Description |";
echo " **Exp. - Band** |";
echo " **Distinct Peptides** |";
echo " **Distinct Spectra** |";
echo " **Method** |";
echo "
";
$i=1;
$arr = file("test.csv");
foreach ($arr as $line) {
$count = $i;
list($protein,$aliasse,$description,$bandI,$distinct\_pepI,$distinct\_seqI,$bandII,$distinct\_pepII,$distinct\_seqII,$method) = split(',', $line);
if($bandII != "" && $bandI == ""){
if($i == 1){
echo "|";
echo " **$protein** $description |";
echo " "; if($bandI == ""){} else{ echo"$bandI";} if($bandI != "" && $bandII != ""){echo " ";} if($bandII == ""){} else{ echo"$bandII";} echo" |";
echo " "; if($bandI == ""){} else{ echo" $distinct\_pepI";} if($bandI != "" && $bandII != ""){echo " ";} if($bandII ==""){} else{echo " $distinct\_pepII";} echo " |";
echo " "; if($bandI == ""){} else{ echo" $distinct\_seqI";} if($bandI != "" && $bandII != ""){echo " ";} if($bandII ==""){} else{echo " $distinct\_seqII";} echo " |";
echo " $method |";
echo "
";
}else{
echo "|";
echo " "; if($bandI == ""){} else{ echo"$bandI";} if($bandI != "" && $bandII != ""){echo " ";} if($bandII == ""){} else{ echo"$bandII";} echo" |";
echo " "; if($bandI == ""){} else{ echo" $distinct\_pepI";} if($bandI != "" && $bandII != ""){echo " ";} if($bandII ==""){} else{echo " $distinct\_pepII";} echo " |";
echo " "; if($bandI == ""){} else{ echo" $distinct\_seqI";} if($bandI != "" && $bandII != ""){echo " ";} if($bandII ==""){} else{echo " $distinct\_seqII";} echo " |";
echo " $method |";
echo "
";
if($i == 4){
echo "|  |
";
$i=0;
}
}
$i++;
}
}
echo "

";
?>
php } ?
php include("layout/footer.php"); ?
