# Supplementary material for: 2DB: a Proteomics database for storage, analysis, presentation, and retrieval of information from mass spectrometric experiments
Source: BMC Bioinformatics. 2008 Jul 7;9:302. doi: 10.1186/1471-2105-9-302 (PMC2475538; doi:10.1186/1471-2105-9-302)
Supplement: Additional file 1 — All files needed to run and further develop the database application as well as the user manual have been bundled into one zip file which can be downloaded from biomedcentral here. Due to constant upgrading of the system, it may be beneficial to check for the latest version on our website [12]. All the sources and additional installation files. [file 1471-2105-9-302-S1.zip › quantSpecCount.php]

2DB - Quantification by spectral count
php
include("layout/menu.php");
?

## Quantification by spectral count

php
//////////////////////////////////////////////////
// Quantify Experiments by Spectral Count //
//////////////////////////////////////////////////
if (!isset ($\_COOKIE["login"])){
echo Warning("bad", "You have to be logged in to use this funktion!");
}
else{
echo "<form action=\"quantSpecCount.php\" method=\"post\"\n";
echo "

\n";
echo "|  |  |
| --- | --- |
|\n";
echo " **Choose Experiments:** |\n";
echo "  |\n";
echo "
\n";
echo "|\n";
echo "  |\n";
echo "
\n";
echo "|\n";
echo " \n"; echo "\n"; $rs = GetResultTableSQL("SELECT ID, Name, Date FROM Separations"); if(!$rs){ echo"----------\n"; }else{ for($i=0; $i$row[1] ($datum)\n"; } } echo "\n"; echo " |\n";
echo " \n"; echo "\n"; if(!$rs){ echo"----------\n"; }else{ $rs = GetResultTableSQL("SELECT ID, Name, Date FROM Separations"); for($i=0; $i$row[1] ($datum)\n"; } } echo "\n"; echo " |\n";
echo "
\n";
echo "|\n";
echo " "; //echo " **Quantification Parameters:** \n"; //echo " Sum Count \n"; //echo " Maximum Count \n"; //echo " Minimum Count \n"; //echo " Average Count \n"; //echo " \n"; echo "  \n"; echo " \n"; echo " |\n";
echo "
\n";
echo "

\n";
echo "\n";
}
?>
php
if(isset($csv)){
echo "<hr";
echo "";
echo "**Choose Value-Separator:**  
";
echo " semicolon";
echo " comma";
echo " tabstop";
echo "";
echo " (custom)";
echo "";
echo "";
echo "  ";
echo "";
}
if(isset($http)){
$time\_start = time();
$rs = GetResultTableSQL("Select Separations.Name FROM Separations WHERE ID = '$sepID1'");
$sepName1 = $rs[0];
echo "

---

";
echo "

## Quantification for $sepName1[0]"; if($sepID1 != $sepID2){ $rs = GetResultTableSQL("Select Separations.Name FROM Separations WHERE ID = '$sepID2'"); $sepName2 = $rs[0]; echo " and $sepName2[0]"; } echo "

";
echo "$sepName1[0]:    ";
if($sepID1 != $sepID2){
echo "$sepName2[0]:   
";
}
echo "  
";
$sql="";
$sql.="###################################PepNumAvg";
$sql.="## Define all proteins for";
$sql.="## the first exp.";
$sql.="###################################";
$sql.="DROP TABLE IF EXISTS AllProts1";
mysql\_query($sql);
$sql="";
$sql.="CREATE TEMPORARY TABLE AllProts1 (";
$sql.="SELECT \* FROM (";
$sql.="SELECT ";
$sql.="f.Name AS frac, p.ID AS pid ";
$sql.="FROM ";
$sql.="( ( ( ( ( (";
$sql.="Separations AS s INNER JOIN Fractionations AS f ON s.ID=f.SeparationID) ";
$sql.="INNER JOIN Experiments AS e ON e.FractionationID=f.ID) ";
$sql.="INNER JOIN Identifications AS i ON i.ExperimentID=e.ID) ";
$sql.="INNER JOIN Peptides AS pep ON pep.ID=i.PeptideID) ";
$sql.="INNER JOIN PepProt AS pp ON pp.PeptideID=pep.ID) ";
$sql.="INNER JOIN Proteins AS p ON p.ID=pp.ProteinID) ";
$sql.="INNER JOIN Software AS so ON so.ID = i.SoftwareID ";
$sql.="WHERE ";
$sql.="s.ID='$sepID1' ";
$sql.="GROUP BY ";
$sql.="frac,pid ";
$sql.="HAVING ";
$sql.="count(DISTINCT pep.ID) >='2' ";
$sql.="UNION ";
$sql.="";
$sql.="SELECT ";
$sql.="f.Name AS frac, p.ID AS pid ";
$sql.="FROM ";
$sql.="( ( ( ( ( (";
$sql.="Separations AS s INNER JOIN Fractionations AS f ON s.ID=f.SeparationID) ";
$sql.="INNER JOIN Experiments AS e ON e.FractionationID=f.ID) ";
$sql.="INNER JOIN Identifications AS i ON i.ExperimentID=e.ID) ";
$sql.="INNER JOIN Peptides AS pep ON pep.ID=i.PeptideID) ";
$sql.="INNER JOIN PepProt AS pp ON pp.PeptideID=pep.ID) ";
$sql.="INNER JOIN Proteins AS p ON p.ID=pp.ProteinID) ";
$sql.="INNER JOIN Software AS so ON so.ID = i.SoftwareID ";
$sql.="WHERE ";
$sql.="s.ID='$sepID1' AND so.ID='2' ";
$sql.="GROUP BY ";
$sql.="frac,pid ";
$sql.="HAVING ";
$sql.="count(DISTINCT pep.ID) >='1' ";
$sql.=") AS tmp) ";
mysql\_query($sql) or die("Query1".mysql\_error());
$sql="";
$sql.="###################################";
$sql.="## Define all proteins for";
$sql.="## the sec. exp.";
$sql.="###################################";
$sql.="DROP TABLE IF EXISTS AllProts2";
mysql\_query($sql);
$sql="";
$sql.="CREATE TEMPORARY TABLE AllProts2 (";
$sql.="SELECT \* FROM (";
$sql.="SELECT ";
$sql.="f.Name AS frac, p.ID AS pid ";
$sql.="FROM ";
$sql.="( ( ( ( ( (";
$sql.="Separations AS s INNER JOIN Fractionations AS f ON s.ID=f.SeparationID) ";
$sql.="INNER JOIN Experiments AS e ON e.FractionationID=f.ID) ";
$sql.="INNER JOIN Identifications AS i ON i.ExperimentID=e.ID) ";
$sql.="INNER JOIN Peptides AS pep ON pep.ID=i.PeptideID) ";
$sql.="INNER JOIN PepProt AS pp ON pp.PeptideID=pep.ID) ";
$sql.="INNER JOIN Proteins AS p ON p.ID=pp.ProteinID) ";
$sql.="INNER JOIN Software AS so ON so.ID = i.SoftwareID ";
$sql.="WHERE ";
$sql.="s.ID='$sepID2' ";
$sql.="GROUP BY ";
$sql.="frac,pid ";
$sql.="HAVING ";
$sql.="count(DISTINCT pep.ID) >='2' ";
$sql.="UNION ";
$sql.="SELECT ";
$sql.="f.Name AS frac, p.ID AS pid ";
$sql.="FROM ";
$sql.="( ( ( ( ( (";
$sql.="Separations AS s INNER JOIN Fractionations AS f ON s.ID=f.SeparationID) ";
$sql.="INNER JOIN Experiments AS e ON e.FractionationID=f.ID) ";
$sql.="INNER JOIN Identifications AS i ON i.ExperimentID=e.ID) ";
$sql.="INNER JOIN Peptides AS pep ON pep.ID=i.PeptideID) ";
$sql.="INNER JOIN PepProt AS pp ON pp.PeptideID=pep.ID) ";
$sql.="INNER JOIN Proteins AS p ON p.ID=pp.ProteinID) ";
$sql.="INNER JOIN Software AS so ON so.ID = i.SoftwareID ";
$sql.="WHERE ";
$sql.="s.ID='$sepID2' AND so.ID='2' ";
$sql.="GROUP BY ";
$sql.="frac,pid ";
$sql.="HAVING ";
$sql.="count(DISTINCT pep.ID) >='1' ";
$sql.=") AS tmp1 ) ";
mysql\_query($sql) or die("Query2".mysql\_error());
$sql="DROP TABLE IF EXISTS ja12";
mysql\_query($sql);
$sql="";
$sql.="CREATE TEMPORARY TABLE ja12 (";
$sql.="SELECT ";
$sql.="a1.pid ";
$sql.="FROM ";
$sql.="AllProts1 AS a1 INNER JOIN AllProts2 AS a2 ON a1.pid=a2.pid";
$sql.=")";
mysql\_query($sql) or die("Query3".mysql\_error());
$sql="DROP TABLE IF EXISTS res1";
mysql\_query($sql);
$sql="";
$sql.="CREATE TEMPORARY TABLE res1 (\n";
$sql.="SELECT DISTINCT sep AS Separation, pid AS ProteinID \n";
$sql.=",COUNT(DISTINCT frac) AS FracNum \n";
$sql.=",SUM(SumPepNum) AS PepSum ,MAX(SumPepNum) AS PepNumBestFrac \n";
$sql.=",AVG(SumPepNum) AS PepNumAvg ,MIN(SumPepNum) AS PepNumMin \n";
$sql.=",SUM(SumSpecNum) AS SpecSum ,MAX(SumSpecNum) AS SpecNumBestFrac \n";
$sql.=",AVG(SumSpecNum) AS SpecNumAvg ,MIN(SumSpecNum) AS SpecNumMin \n";
$sql.="FROM \n";
$sql.="( \n";
$sql.="SELECT \n";
$sql.="f.Name AS frac, p.ID AS pid, s.Name AS sep \n";
$sql.=",so.ID AS soft, pep.Sequence AS seq, i.Spectrum AS spec \n";
$sql.=",COUNT(DISTINCT pep.Sequence) AS SumPepNum, COUNT(DISTINCT i.Spectrum) AS SumSpecNum \n";
$sql.="FROM \n";
$sql.="( ( ( ( ( ( \n";
$sql.="Separations AS s INNER JOIN Fractionations AS f ON s.ID=f.SeparationID) \n";
$sql.="INNER JOIN Experiments AS e ON e.FractionationID=f.ID) \n";
$sql.="INNER JOIN Identifications AS i ON i.ExperimentID=e.ID) \n";
$sql.="INNER JOIN Peptides AS pep ON pep.ID=i.PeptideID) \n";
$sql.="INNER JOIN PepProt AS pp ON pp.PeptideID=pep.ID) \n";
$sql.="INNER JOIN Proteins AS p ON p.ID=pp.ProteinID) \n";
$sql.="INNER JOIN Software AS so ON so.ID = i.SoftwareID \n";
$sql.="WHERE \n";
$sql.="s.ID='$sepID1' \n";
$sql.="AND ( \n";
$sql.="p.ID IN (SELECT pid FROM ja12) \n";
$sql.=") \n";
$sql.="GROUP BY \n";
$sql.="p.ID, f.ID \n";
$sql.=") AS temp \n";
$sql.="GROUP BY \n";
$sql.="pid \n";
$sql.="#res1 table \n";
$sql.=") \n";
mysql\_query($sql) or die("Query4".mysql\_error());
$sql="DROP TABLE IF EXISTS TABLE res2";
mysql\_query($sql);
$sql="";
$sql.="CREATE TEMPORARY TABLE res2 (\n";
$sql.="SELECT DISTINCT sep AS Separation, pid AS ProteinID \n";
$sql.=",COUNT(DISTINCT frac) AS FracNum \n";
$sql.=",SUM(SumPepNum) AS PepSum ,MAX(SumPepNum) AS PepNumBestFrac \n";
$sql.=",AVG(SumPepNum) AS PepNumAvg ,MIN(SumPepNum) AS PepNumMin \n";
$sql.=",SUM(SumSpecNum) AS SpecSum ,MAX(SumSpecNum) AS SpecNumBestFrac \n";
$sql.=",AVG(SumSpecNum) AS SpecNumAvg ,MIN(SumSpecNum) AS SpecNumMin \n";
$sql.="FROM \n";
$sql.="(\n";
$sql.="SELECT \n";
$sql.="f.Name AS frac, p.ID AS pid, s.Name AS sep \n";
$sql.=",so.ID AS soft, pep.Sequence AS seq, i.Spectrum AS spec \n";
$sql.=",COUNT(DISTINCT pep.Sequence) AS SumPepNum, COUNT(DISTINCT i.Spectrum) AS SumSpecNum \n";
$sql.="FROM \n";
$sql.="( ( ( ( ( (\n";
$sql.="Separations AS s INNER JOIN Fractionations AS f ON s.ID=f.SeparationID) \n";
$sql.="INNER JOIN Experiments AS e ON e.FractionationID=f.ID) \n";
$sql.="INNER JOIN Identifications AS i ON i.ExperimentID=e.ID) \n";
$sql.="INNER JOIN Peptides AS pep ON pep.ID=i.PeptideID) \n";
$sql.="INNER JOIN PepProt AS pp ON pp.PeptideID=pep.ID) \n";
$sql.="INNER JOIN Proteins AS p ON p.ID=pp.ProteinID) \n";
$sql.="INNER JOIN Software AS so ON so.ID = i.SoftwareID \n";
$sql.="WHERE \n";
$sql.="s.ID='$sepID2' \n";
$sql.="AND (\n";
$sql.="p.ID IN (SELECT pid FROM ja12) \n";
$sql.=") \n";
$sql.="GROUP BY \n";
$sql.="p.ID, f.ID \n";
$sql.=") AS temp1 \n";
$sql.="GROUP BY \n";
$sql.="pid \n";
$sql.="#res2 table \n";
$sql.=") \n";
mysql\_query($sql) or die("Query5".mysql\_error());
$sql="SELECT
res1.ProteinID
,p.Name,p.Description
,res1.FracNum ,res2.FracNum
,res1.PepSum, res2.PepSum ,(res1.PepSum/res2.PepSum) AS SumRatio
,res1.PepNumBestFrac ,res2.PepNumBestFrac ,(res1.PepNumBestFrac/res2.PepNumBestFrac) AS BestRatio
,res1.PepNumAvg ,res2.PepNumAvg ,(res1.PepNumAvg/res2.PepNumAvg) AS AvgRatio
,res1.PepNumMin ,res2.PepNumMin
,res1.SpecSum ,res2.SpecSum
,res1.SpecNumBestFrac ,res2.SpecNumBestFrac
,res1.PepNumAvg ,res2.PepNumAvg
,res1.SpecNumMin ,res2.SpecNumMin
FROM
(
res1 INNER JOIN res2 ON res1.ProteinID=res2.ProteinID)
INNER JOIN Proteins AS p ON p.ID=res1.ProteinID
ORDER BY p.Name asc";
echo "

\n";
echo "|  |  |  |  |  |  |  |  |  |  |  |
| --- | --- | --- | --- | --- | --- | --- | --- | --- | --- | --- |
|\n";
echo " **Name/Description** |\n";
echo " **Data** |\n";
echo " **FracNum** |\n";
echo " **PepSum** |\n";
echo " **PepNumBestFrac** |\n";
echo " **PepNumAvg** |\n";
echo " **PepNumMin** |\n";
echo " **SpecSum** |\n";
echo " **SpecNumBestFrac** |\n";
echo " **SpecNumAvg** |\n";
echo " **SpecNumMin** |\n";
echo "
\n";
$rs = GetResultTableSQL($sql);
if(!$rs){ echo "No results";}
else{
for($i=0; $i\n";
echo " **".GetLink($row[0])."** $row[2] |\n"; // ID // Name // Description
echo " $sepName1[0] |\n"; // Data
echo " $row[3] |\n"; // FracNum
echo " $row[5] |\n"; // PepSum
echo " $row[8] |\n"; // PepNumBestFrac
echo " ".round($row[11],2)." |\n"; //PepNumAvg
echo " $row[14] |\n"; //PepNumMin
echo " $row[16] |\n"; //SpecSum
echo " $row[18] |\n"; //SpecNumBestFrac
echo " ".round($row[20],2)." |\n"; //SpecNumAvg
echo " $row[22] |\n"; //SpecNumMin
echo "\n|\n";
if($sepID1 != $sepID2){
echo " $sepName2[0] |\n"; // Data
echo " $row[4] |\n"; // FracNum\_1
echo " $row[6] |\n"; // PepSum\_1
echo " $row[9] |\n"; //PepNumBestFrac\_1
echo " ".round($row[12],2)." |\n"; //PepNumAvg\_1
echo " $row[15] |\n"; //PepNumMin\_1
echo " $row[17] |\n"; //SpecSum\_1
echo " $row[19] |\n"; //SpecNumBestFrac\_1
echo " ".round($row[21],2)." |\n"; //SpecNumAvg
echo " $row[23] |\n"; //SpecNumMin\_1
echo "
\n|\n";
echo " Ratio |\n"; // Data
echo "  |\n"; // FracNum\_empty
echo " $row[7] |\n"; // SumRatio
echo " $row[10] |\n"; // BestRatio
echo " ".round($row[13],2)." |\n"; //AvgRatio
echo "  |\n"; // PepNumMin\_empty
echo "  |\n"; //SpecSum\_empty
echo "  |\n"; //SpecNumBestFrac\_empty
echo "  |\n"; //PepNumAvg\_empty
echo "  |\n"; //SpecNumMin\_empty
echo "
\n";
}
if($sepID1 != $sepID2){
echo "|  |
\n"; // wenn die beiden letzten je +2
}
if($sepID1 == $sepID2){
echo "|  |
\n";
}
}
}
echo "

";
echo "  
";
$time\_end = time();
echo "It took ".($time\_end - $time\_start)." sec to generate this quantification.";
}
?>
php include("layout/footer.php"); ?
