# Supplementary material for: 2DB: a Proteomics database for storage, analysis, presentation, and retrieval of information from mass spectrometric experiments
Source: BMC Bioinformatics. 2008 Jul 7;9:302. doi: 10.1186/1471-2105-9-302 (PMC2475538; doi:10.1186/1471-2105-9-302)
Supplement: Additional file 1 — All files needed to run and further develop the database application as well as the user manual have been bundled into one zip file which can be downloaded from biomedcentral here. Due to constant upgrading of the system, it may be beneficial to check for the latest version on our website [12]. All the sources and additional installation files. [file 1471-2105-9-302-S1.zip › resultHTMLoutput.php]

2DB - Compare Experiments
php
include("layout/menu.php");
$sql = "Select Separations.Name from Separations Where Separations.ID = '$sep' Limit 1";
$rs = GetResultTableSQL($sql);
$experimentname = $rs[0];
?

## Significant Result for php echo "$experimentname[0]"; ?

php
//////////////////////////////////////////////////
// Show the result list as html //
//////////////////////////////////////////////////
if (!isset ($\_COOKIE["login"])){
echo "<div style=\"padding:4px; border-color:#FF0000; border-width:1px; border-style:solid;\"**!** You have to be logged in to use this funktion!  
\n";
}
else{
?>

php
function SpecCount($sepid, $fracid, $protid) {
$sql.="SELECT COUNT(DISTINCT i.Spectrum) FROM ";
$sql.="( ( ( ";
$sql.="(SELECT Fractionations.ID AS fid, s.ID AS sid FROM ";
$sql.="(SELECT ID FROM Separations ";
$sql.="WHERE Separations.ID='$sepid') ";
$sql.="AS s ";
$sql.="INNER JOIN Fractionations ON Fractionations.SeparationID=s.ID ";
$sql.="WHERE Fractionations.ID='$fracid') ";
$sql.="AS f ";
$sql.="INNER JOIN Experiments AS e ON e.FractionationID=f.fid) ";
$sql.="INNER JOIN Identifications AS i ON i.ExperimentID=e.ID ";
$sql.="INNER JOIN Peptides AS pep ON pep.ID=i.PeptideID) ";
$sql.="INNER JOIN PepProt AS pp ON pp.PeptideID=pep.ID) ";
$sql.="INNER JOIN Proteins AS p ON p.ID=pp.ProteinID ";
$sql.="WHERE p.ID = $protid";
$rs = GetResultTableSQL($sql);
return($rs[0][0]);
}
function SeqCov($sepid, $fracid, $protid) {
$sql.="SELECT DISTINCT pep.Sequence FROM ";
$sql.="( ( ";
$sql.="(SELECT Fractionations.ID AS fid, s.ID AS sid FROM ";
$sql.="(SELECT ID FROM Separations ";
$sql.="WHERE Separations.ID='$sepid') ";
$sql.="AS s ";
$sql.="INNER JOIN Fractionations ON Fractionations.SeparationID=s.ID ";
$sql.="WHERE Fractionations.ID='$fracid') ";
$sql.="AS f ";
$sql.="INNER JOIN Experiments AS e ON e.FractionationID=f.fid) ";
$sql.="INNER JOIN Identifications AS i ON i.ExperimentID=e.ID ";
$sql.="INNER JOIN Peptides AS pep ON pep.ID=i.PeptideID) ";
$sql.="INNER JOIN PepProt AS pp ON pp.PeptideID=pep.ID ";
$sql.="WHERE pp.ProteinID='$protid' ORDER BY LENGTH(pep.Sequence) DESC";
$peps = GetResultTableSQL($sql);
$prots=GetResultTableSQL("SELECT s.Sequence FROM Proteins AS p INNER JOIN Sequences AS s ON s.ID=p.SequenceID WHERE p.ID='$protid'");
$pepsLen = 0;
$cleanPeps = array();
$ct=0;
for($i=0; $i<count($peps); $i++) {
$found = false;
for($s=0; $s<count($cleanPeps); $s++) {
if(strpos($peps[$i][0],$cleanPeps[$s][0]) !== false)
$found = true;
}
if(!$found)
$cleanPeps[$ct++]=$peps[$i];
}
for($i=0; $i<count($cleanPeps); $i++)
$pepsLen += strlen($cleanPeps[$i][0]);
$protLen = strlen($prots[0][0]);
if($protLen == 0)
$protLen = 1;
$seqcov = $pepsLen/$protLen;
$seqcov = round($seqcov,3);
return($seqcov);
}
function EchoArr($arr,$separator) {
$row = "";
$row .= "<tr";
for($i=0; $i" . $arr[$i] . "";
}
$row .= "";
//$row = substr($row,0,strlen($row)-1);
echo"$row";
}
function PrintProt($protarr,$fracid,$pid) {
for($i=0; $i");
//Thresholds have been retrieved
}
if(!$thresholds) {
die("Problem with thresholds  
");
}
//Here we should output the headings of the table.
$data = array();
$ct = 0;
$data[$ct++] = "**Band/Spot**";
$data[$ct++] = "**Protein**";
$data[$ct++] = "**Description**";
$data[$ct++] = "**Spectral Count**";
$data[$ct++] = "**Distinct Peptides**";
$data[$ct++] = "**Sequence Coverage (%)**";
$softs = GetResultTableSQL("SELECT \* FROM Software WHERE UPPER(Name) != UPPER('2db')");
for($i=0; $i  
(normal/intron split)";
}
//Get all Fractionations from the table in question
$sql = "SELECT Distinct ID,Name FROM Fractionations WHERE SeparationID = '$sepid'";
$franz = GetResultTableSQL($sql);
//////////////////////////////////////////////////
function naturalsort($item1, $item2) {
return strnatcmp($item1[1], $item2[1]);
}
usort($franz, "naturalsort");
///////////////////////////////////////////////
EchoArr($data,$separator);
//The header fields are now printed to file.
$shownProts = array();
//echo"Count: " . count($franz) . "\r\n";
for($fracs=0; $fracs0)
$sql.="WHERE $whereRestraints ";
if(strlen($restraints) > 0)
$sql.="GROUP BY p.ID HAVING $restraints ";
$rs=GetResultTableSQL($sql);
if($rs) {
//loop throught the available proteins below
for($o=0; $o".$franz[$fracs][1].""; //Fractionation name and Link
$proteins = $prot[1];
$pr=GetResultTable("Alias","Aliases","ProteinID=".$prot[0]);
for($z=0; $z"; //Spectral count
$ret = mysql\_query("DELETE FROM AllProts WHERE pid='$prot[0]'");
$sql="";
$sql.="SELECT DISTINCT pep.ID FROM ";
$sql.="( ( ";
$sql.="(SELECT Fractionations.ID AS fid, s.ID AS sid FROM ";
$sql.="(SELECT ID FROM Separations ";
$sql.="WHERE Separations.ID='$sepid') AS s ";
$sql.="INNER JOIN Fractionations ON Fractionations.SeparationID=s.ID ";
$sql.="WHERE Fractionations.ID='$fracid') ";
$sql.="AS f ";
$sql.="INNER JOIN Experiments AS e ON e.FractionationID=f.fid) ";
$sql.="INNER JOIN Identifications AS i ON i.ExperimentID=e.ID ";
$sql.="INNER JOIN Peptides AS pep ON pep.ID=i.PeptideID) ";
$sql.="INNER JOIN (SELECT \* FROM PepProt WHERE ProteinID='$prot[0]') ";
$sql.="AS pp ON pp.PeptideID=pep.ID ";
$peps = GetResultTableSQL($sql);
$pepNum = count($peps);
$data[$ct++] = "".$pepNum.""; //Distinct peptides
$num = SeqCov($sepid,$fracid,$prot[0]) \* 100;
settype($num,"integer");
if($separator == "g")
$num = str\_replace(".",",",$num);
$data[$ct++] = " ".$num ."%"; //Sequence coverage
for($s=0; $s"; //Distinct peptides (softbased)
}
if(PrintProt($shownProts,$fracid,$prot[0])) {
EchoArr($data,$separator);
$shownProts[count($shownProts)] = array($fracid,$prot[0]);
}
}
}
}
}
}
?>

php include("layout/footer.php"); ?
