# Supplementary material for: 2DB: a Proteomics database for storage, analysis, presentation, and retrieval of information from mass spectrometric experiments
Source: BMC Bioinformatics. 2008 Jul 7;9:302. doi: 10.1186/1471-2105-9-302 (PMC2475538; doi:10.1186/1471-2105-9-302)
Supplement: Additional file 1 — All files needed to run and further develop the database application as well as the user manual have been bundled into one zip file which can be downloaded from biomedcentral here. Due to constant upgrading of the system, it may be beneficial to check for the latest version on our website [12]. All the sources and additional installation files. [file 1471-2105-9-302-S1.zip › search.php]

2Db - Search
php include("layout/menu.php"); ?

## Search

php echo "<form action='" . $\_SERVER['PHP\_SELF'] . "' method='POST'"; ?>
 
 

  
  

php
///////////////////////////////////////////////////////////////////////
// Initialize Colors //
///////////////////////////////////////////////////////////////////////
$headcolor = "#FFFF80"; // Results
$protcolor = "#ffffff"; // Proteine
$pepcolor = "#ffffff"; // Peptide
$falsecolor = "#cccccc"; // No Results
///////////////////////////////////////////////////////////////////////
// Initialize variables //
///////////////////////////////////////////////////////////////////////
$query = trim($\_POST['query']);
$len = strlen($query);
if($len <= 0) {
$query=trim($\_GET['query']);
$len = strlen($query);
}
if(!isset($\_GET['limit']))
$limit = 20;
else {
if($limit = -1)
$limit = 999999;
else
$limit = $\_POST['limit'];
}
$seqQuery = strtoupper($query); //For fast 'case insensitve' sequence search
$uid = $tab[1];
//////Initialize plugins//////////
$plugins = array();
$verz=opendir ('plugins');
$i = 0;
while ($file = readdir ($verz)) {
if((strpos($file,".")  2) && $file != "index.php"){
$function = explode(".",$file);
$plugins[$i++] = array($file,$function[0]);
}
}
closedir($verz);
///////////////////////////////////////////////////////////////////////
// Initialized variables //
///////////////////////////////////////////////////////////////////////
function ShowTable($rawTab) {
if(!$rawTab)
return(false);
$ct = count($rawTab);
if($ct <= 0)
return(false);
echo"

";
for($o=0; $o<$ct; $o++) {
$row = $rawTab[$o];
echo"|  |
| --- |
|";
$ict=count($row);
for($i=0; $i<$ict; $i++) {
echo" " . $row[$i] . " |";
}
echo"
";
if($o > 3) {
echo"| ... |
";
break;
}
}
echo"

";
return(true);
}
if(isset($\_POST['retrieve']) || isset($\_GET['query'])AND $query !="") {
//$rs = ConDB($dbname);
//////////////////////////////////////////////////////////////////
// ProteinIDs //
//////////////////////////////////////////////////////////////////
if($len >= 3) {
$sql="";
$sql.="SELECT p.ID, s.Sequence, p.Name FROM ";
$sql.="(SELECT \* FROM Proteins WHERE UPPER(Name) LIKE UPPER('%$query%')) AS p ";
$sql.="INNER Join Sequences AS s ON s.ID=p.SequenceID ";
$sql.="UNION ALL ";
$sql.="SELECT a.ProteinID, s2.Sequence, a.Alias FROM ";
$sql.="( ";
$sql.="(SELECT \* FROM Aliases WHERE UPPER(Alias) LIKE UPPER('%$query%')) AS a ";
$sql.="INNER JOIN Proteins AS p2 ON p2.ID=a.ProteinID ) ";
$sql.="INNER JOIN Sequences AS s2 ON s2.ID=p2.SequenceID ";
$rs = GetResultTableSQL($sql);
//$rs = GetResultTable("DISTINCT Proteins.ID, Sequence, Proteins.Name, Aliases.Alias","(Proteins INNER JOIN Aliases ON Aliases.ProteinID=Proteins.ID) INNER JOIN Sequences ON Sequences.ID=Proteins.SequenceID","(UPPER(Name) LIKE UPPER('%$query%')) OR (UPPER(Alias) LIKE UPPER('%$query%')) ORDER BY Proteins.ID");
if($rs) {
$results = count($rs);
echo"";
echo"\n";
echo"Found $results **Protein(s)** that match your query. ";
if($results>$limit)
echo" Show all  
\n";
else
echo"  
";
echo"";
echo"

\n";
echo"

\n";
echo"| JGI-TID | Sequence |
| --- | --- |
\n";
$sLim = $limit;
if($sLim > $results)
$sLim = $results;
for($o=0; $o<$sLim; $o++) {
$bgcolor = ($o % 2) ? "#ff0000" : "#ffffff";
$row = $rs[$o];
$rs2 = GetResultTable("\*","Locations","ProteinID=$row[0]");
if(!$rs2) continue;
$rs2=$rs2[0];
$rs3 = GetResultTable("\*","Sources","ID=$rs2[5]");
if(!$rs3) continue;
$rs3 = $rs3[0];
echo"|\n";
echo" " . GetLink($row[0]) . "  \n"; /////////////////use plugins//////////////// echo ""; echo ""; echo "Use Tool:\n"; echo "----------\n"; echo "Set Description\n"; echo ">FASTA\n"; for($g=0; $g" . $plugins[$g][1] . "\n"; } echo "\n"; echo " |\n";
/////////////////\use plugins////////////////
$ts = SplitString($row[1],60,false);
echo" $ts |\n";
//echo" " . $row[1] . " |
\n";
$sql="CREATE TEMPORARY TABLE pidt ";
$sql.="SELECT s.Name AS sn, s.ID as sid, f.ID as fid, s.Image FROM ";
$sql.="( ( ( ( ( ( ( (";
$sql.="(SELECT p.ID AS pid FROM Proteins AS p WHERE p.ID='$row[0]') AS prot ";
$sql.="INNER JOIN PepProt AS pp ON pp.ProteinID=prot.pid) ";
$sql.="INNER JOIN Peptides AS pep ON pep.ID=pp.PeptideID) ";
$sql.="INNER JOIN Identifications AS i ON i.PeptideID=pep.ID) ";
$sql.="INNER JOIN Experiments AS e ON e.ID=i.ExperimentID) ";
$sql.="INNER JOIN Fractionations AS f ON f.ID=e.FractionationID) ";
$sql.="INNER JOIN Separations AS s on s.ID=f.SeparationID) ";
$sql.="LEFT JOIN Access AS a ON a.RowID=s.ID) ";
$sql.="LEFT JOIN Members AS m ON a.GroupID=m.GroupID) ";
$sql.="LEFT JOIN Groups AS g ON g.ID=m.GroupID ";
$sql.="WHERE (m.UserID='$uid' AND a.Table='Separations') ";
$sql.="OR s.OperatorID='$uid' OR g.Name='Guests'";
mysql\_query($sql);
$locs = GetResultTableSQL("SELECT DISTINCT sn,sid,Image FROM pidt");
$seps = count($locs);
if($seps > 0 && $locs != false) {
echo"| \n"; echo"\n"; echo"\n"; echo"Found $seps **Separation(s)** that contains this protein. \n"; echo""; echo" \n"; echo"  "; for($s=0; $s<$seps; $s++) { $matchedFracs=GetResultTableSQL("SELECT ID,Name FROM Fractionations WHERE ID IN (SELECT fid FROM pidt WHERE sid=" . $locs[$s][1] . ")"); //Show all the separations that contain the query along with the fractionations that contain the query echo "|  |  | | --- | --- | | "; $imgp = "gelimages/thumbs/" . $locs[$s][2]; if(!file\_exists($imgp) || is\_dir($imgp)) $imgp = "gelimages/thumbs/no\_picture.jpg"; $size = getimagesize ($imqp); $max\_width = 50; // maximum width $max\_height = 50; // maximum width $width = $size[0]; $height = $size[1]; if($width==0) $width=1; if($height==0) $height=1; $ratio = $max\_width / $width; $test = $height \* $ratio; if($test > $max\_height){ $ratio = $max\_height / $height; } $n\_width = $width \* $ratio; $n\_height = $height \* $ratio; echo " "; echo "" . $locs[$s][0] . " | "; for($f=0; $f" . $matchedFracs[$f][1] . ""; else echo"" . $matchedFracs[$f][1] . ", "; } echo" | "; } echo" \n"; echo" |
\n";
}
}
echo"

\n";
echo"

  
\n";
}
}
if($len >= 4) {
//////////////////////////////////////////////////////////////////
// Sequences //
//////////////////////////////////////////////////////////////////
$rs = GetResultTable("Sequences.ID,Proteins.ID,Proteins.Name,Sequence","Sequences INNER JOIN Proteins ON Proteins.SequenceID=Sequences.ID","Sequence LIKE '%$seqQuery%'");
if($rs) {
$results = count($rs);
echo"";
echo"\n";
echo"Found $results **Sequence(ses)** that match your query.";
if($results>$limit)
echo" Show all  
\n";
else
echo"  
";
echo"";
echo"

\n";
echo"

\n";
$sLim = $limit;
if($sLim > $results)
$sLim = $results;
for($o=0; $o<$sLim; $o++) {
$bgcolor = ($o % 2) ? "#FFFFCC" : "#ffffff";
$row = $rs[$o];
$rs2 = GetResultTable("\*","Locations","ProteinID=$row[1]");
if(!$rs2) continue;
$rs2=$rs2[0];
$rs3 = GetResultTable("\*","Sources","ID=$rs2[5]");
if(!$rs3) continue;
$rs3 = $rs3[0];
echo"|  |  |
| --- | --- |
|\n";
echo" " . GetLink($row[0]) . "  "; //echo "copy |\n";
/////////////////use plugins////////////////
echo "";
echo "";
echo "Use Tool:\n";
echo "----------\n";
echo "Set Description\n";
echo ">FASTA\n";
for($g=0; $g" . $plugins[$g][1] . "\n";
}
echo "\n";
echo "\n";
/////////////////\use plugins////////////////
///////// Sequence Highlight ////////
$search = "/$seqQuery/";
$replace = "$seqQuery";
$str = preg\_replace($search, $replace, $row[3]);
$ts = SplitString($str,60,false);
echo" $ts |\n";
echo"
\n";
$sql="CREATE TEMPORARY TABLE seid ";
$sql.="SELECT DISTINCT s.Name AS sn, s.id AS sid, f.ID AS fid, Image FROM ";
$sql.="( ( ( ( ( ( ( (";
$sql.="(SELECT p.ID AS pid FROM Proteins AS p WHERE p.ID='$row[1]') AS prot ";
$sql.="INNER JOIN PepProt AS pp ON pp.ProteinID=prot.pid) ";
$sql.="INNER JOIN Peptides AS pep ON pep.ID=pp.PeptideID) ";
$sql.="INNER JOIN Identifications AS i ON i.PeptideID=pep.ID) ";
$sql.="INNER JOIN Experiments AS e ON e.ID=i.ExperimentID) ";
$sql.="INNER JOIN Fractionations AS f ON f.ID=e.FractionationID) ";
$sql.="INNER JOIN Separations AS s on s.ID=f.SeparationID) ";
$sql.="LEFT JOIN Access AS a ON a.RowID=s.ID) ";
$sql.="LEFT JOIN Members AS m ON a.GroupID=m.GroupID) ";
$sql.="LEFT JOIN Groups AS g ON g.ID=m.GroupID ";
$sql.="WHERE (m.UserID='$uid' AND a.Table='Separations') ";
$sql.="OR s.OperatorID='$uid' OR g.Name='Guests'";
mysql\_query($sql);
$locs = GetResultTableSQL("SELECT DISTINCT sn,sid,Image FROM seid");
$seps = count($locs);
if($seps > 0 && $locs != false) {
echo"| \n"; echo"\n"; echo"\n"; echo"Found $seps **Separation(s)** that contains this sequence/ protein. \n"; echo""; echo" \n"; echo"  "; for($s=0; $s<$seps; $s++) { $matchedFracs=GetResultTableSQL("SELECT DISTINCT ID,Name FROM Fractionations WHERE ID IN (SELECT fid FROM seid WHERE sid=" . $locs[$s][1] . ")"); //Show all the separations that contain the query along with the fractionations that contain the query echo "|  |  | | --- | --- | | "; $imgp = "gelimages/thumbs/" . $locs[$s][2]; if(!file\_exists($imgp) || is\_dir($imgp)) $imgp = "gelimages/thumbs/no\_picture.jpg"; $size = getimagesize ($imqp); $max\_width = 50; // maximum width $max\_height = 50; // maximum width $width = $size[0]; $height = $size[1]; if($width==0) $width=1; if($height==0) $height=1; $ratio = $max\_width / $width; $test = $height \* $ratio; if($test > $max\_height){ $ratio = $max\_height / $height; } $n\_width = $width \* $ratio; $n\_height = $height \* $ratio; echo " "; echo "" . $locs[$s][0] . " | "; for($f=0; $f" . $matchedFracs[$f][1] . ""; else echo"" . $matchedFracs[$f][1] . ", "; } echo" | "; } echo" \n"; echo" |
\n";
}
}
echo"

\n";
echo"

  
\n";
}
}
if($len >= 3) {
//////////////////////////////////////////////////////////////////
// Peptides //
//////////////////////////////////////////////////////////////////
$sql= "";
$sql.="CREATE TEMPORARY TABLE temp ";
$sql.="SELECT DISTINCT s.Name AS sep, f.Name AS frac, pep.pepid AS pepID, pep.pseq AS seq, Image, s.ID AS sid, f.ID AS fid FROM ";
$sql.="( ( ( ( ( (";
$sql.="(SELECT p.ID AS pepid, p.Sequence AS pseq FROM Peptides AS p WHERE p.Sequence LIKE '%$seqQuery%') AS pep ";
$sql.="INNER JOIN Identifications AS i ON i.PeptideID=pep.pepid) ";
$sql.="INNER JOIN Experiments AS e ON e.ID=i.ExperimentID) ";
$sql.="INNER JOIN Fractionations AS f ON f.ID=e.FractionationID) ";
$sql.="INNER JOIN Separations AS s on s.ID=f.SeparationID) ";
$sql.="LEFT JOIN Access AS a ON a.RowID=s.ID) ";
$sql.="LEFT JOIN Members AS m ON a.GroupID=m.GroupID) ";
$sql.="LEFT JOIN Groups AS g ON g.ID=m.GroupID ";
$sql.="WHERE (m.UserID='$uid' AND a.Table='Separations') ";
$sql.="OR s.OperatorID='$uid' OR g.Name='Guests' OR (g.Name='Administrators' and m.UserID='$uid')";
// echo"$sql  
";
$rs = mysql\_query($sql);
$rs = GetResultTable("DISTINCT pepID, seq","temp","");
if($rs) {
$results = count($rs);
echo"";
echo"\n";
echo"Found $results peptide(s) that match your query.";
if($results>$limit)
echo" Show all  
\n";
else
echo"  
";
echo"";
echo"

\n";
echo"

\n";
$sLim = $limit;
if($sLim > $results)
$sLim = $results;
for($o=0; $o<$sLim; $o++) {
$row = $rs[$o];
$rs2 = GetResultTable("\*","SubPepSeqs","PeptideID='$row[0]'");
$intron = "";
if($rs2) {
for($i=0; $i$seqQuery";
$str = preg\_replace($search, $replace, $row[1]);
$ts = SplitString($str,60,false);
echo"|  |  |
| --- | --- |
|\n";
echo" $ts |\n";
echo" $intron |\n";
echo"
\n";
$sql = "SELECT DISTINCT sid,sep,Image FROM temp WHERE pepid='$row[0]'";
//echo"$sql  
";
$matchedSeps=GetResultTableSQL($sql);
//print\_r($matchedSeps);
//echo"  
";
//echo mysql.error();
$seps = count($matchedSeps);
if($seps > 0) {
echo"| \n"; echo"\n"; echo"\n"; echo"Found $seps **Separation(s)** that contains this peptide. \n"; echo""; echo" \n"; echo"  "; for($s=0; $s<$seps; $s++) { $sql = "SELECT DISTINCT fid,frac FROM temp WHERE sid='" . $matchedSeps[$s][0] . "'"; //echo"$sql "; $matchedFracs=GetResultTableSQL($sql); //Show all the separations that contain the query along with the fractionations that contain the query echo "| "; $imgp = "gelimages/thumbs/" . $matchedSeps[$s][2]; if(!file\_exists($imgp) || is\_dir($imgp)) $imgp = "gelimages/thumbs/no\_picture.jpg"; $size = getimagesize ($imqp); $max\_width = 50; // maximum width $max\_height = 50; // maximum width $width = $size[0]; $height = $size[1]; if($width==0) $width=1; if($height==0) $height=1; $ratio = $max\_width / $width; $test = $height \* $ratio; if($test > $max\_height){ $ratio = $max\_height / $height; } $n\_width = $width \* $ratio; $n\_height = $height \* $ratio; echo " "; echo "" . $matchedSeps[$s][1] . " | "; $lnks = ""; for($f=0; $f" . $matchedFracs[$f][1] . ", "; } echo substr($lnks,0,strlen($lnks)-2); echo" | "; } echo" \n"; echo" |
\n";
}
}
echo"

\n";
echo"

  
\n";
}
}
//////////////////////////////////////////////////////////////////
// Separations //
//////////////////////////////////////////////////////////////////
$sql = "";
$sql.="SELECT DISTINCT s.ID, s.Name, s.Image FROM ( ";
$sql.="Separations AS s LEFT JOIN Access AS a ON a.RowID=s.ID) ";
$sql.="LEFT JOIN Members AS m ON m.GroupID=a.GroupID ";
$sql.="WHERE ((m.UserID='$uid' AND a.Table='Separations') OR s.OperatorID='$uid') ";
$sql.="AND UPPER(Name) LIKE UPPER('%$query%')";
$rs = GetResultTableSQL($sql);
if($rs) {
$results = count($rs);
echo"";
echo"\n";
echo"Found $results **Separation(s)** that match your query. ";
if($results>$limit)
echo" Show all  
\n";
else
echo"  
";
echo"";
echo"

\n";
echo"

\n";
$sLim = $limit;
if($sLim > $results)
$sLim = $results;
for($o=0; $o<$sLim; $o++) {
$row = $rs[$o];
echo"|  |  |
| --- | --- |
|  |";
echo" $row[1] |
";
}
echo"

\n";
echo"

  
\n";
}
//////////////////////////////////////////////////////////////////
// Fractionations //
//////////////////////////////////////////////////////////////////
$sql = "";
$sql.="SELECT DISTINCT s.ID, s.Name, f.ID, f.Name FROM ( ( (";
$sql.="Fractionations AS f INNER JOIN ";
$sql.="Separations AS s ON f.SeparationID=s.ID) LEFT JOIN Access AS a ON a.RowID=s.ID) ";
$sql.="LEFT JOIN Members AS m ON m.GroupID=a.GroupID) ";
$sql.="LEFT JOIN Groups AS g ON g.ID = m.GroupID ";
$sql.="WHERE ((m.UserID='$uid' AND a.Table='Separations') OR s.OperatorID='$uid') ";
$sql.="AND UPPER(f.Name) LIKE UPPER('%$query%')";
$rs = GetResultTableSQL($sql);
if($rs) {
$results = count($rs);
echo"";
echo"\n";
echo"Found $results **Fractionation(s)** that match your query. ";
if($results>$limit)
echo" Show all  
\n";
else
echo"  
";
echo"";
echo"

\n";
echo"

\n";
$sLim = $limit;
if($sLim > $results)
$sLim = $results;
for($o=0; $o<$sLim; $o++) {
$row = $rs[$o];
echo"|  |
| --- |
| $row[1] - $row[3] |
";
}
echo"

\n";
echo"

  
\n";
}
//////////////////////////////////////////////////////////////////
// Experiments //
//////////////////////////////////////////////////////////////////
$sql = "";
$sql.="SELECT DISTINCT s.Name, f.Name, e.Name FROM ( ( ( (";
$sql.="Experiments AS e INNER JOIN Fractionations AS f ON f.ID=e.FractionationID) ";
$sql.="INNER JOIN Separations AS s ON f.SeparationID=s.ID) LEFT JOIN Access AS a ON a.RowID=s.ID) ";
$sql.="LEFT JOIN Members AS m ON m.GroupID=a.GroupID) ";
$sql.="LEFT JOIN Groups AS g ON g.ID = m.GroupID ";
$sql.="WHERE ((m.UserID='$uid' AND a.Table='Separations') OR s.OperatorID='$uid') ";
$sql.="AND UPPER(e.Name) LIKE UPPER('%$query%')";
$rs = GetResultTableSQL($sql);
if($rs) {
$results = count($rs);
echo"";
echo"\n";
echo"Found $results **Experiments(s)** that match your query. ";
if($results>$limit)
echo" Show all  
\n";
else
echo"  
";
echo"";
echo"

\n";
echo"

\n";
$sLim = $limit;
if($sLim > $results)
$sLim = $results;
for($o=0; $o<$sLim; $o++) {
$row = $rs[$o];
echo"|  |  |  |
| --- | --- | --- |
| $row[0] | $row[1] | $row[2] |
";
}
echo"

\n";
echo"

  
\n";
}
///////////////////////////////////////////////////////////////////
$sql= "";
$sql.="CREATE TEMPORARY TABLE identi ";
$sql.="SELECT DISTINCT s.Name AS sep, f.Name AS frac, i.Spectrum AS dta, i.PeptideID AS pepID FROM ";
$sql.="( ( ( ( ( ";
$sql.="Identifications AS i ";
$sql.="INNER JOIN Experiments AS e ON e.ID=i.ExperimentID) ";
$sql.="INNER JOIN Fractionations AS f ON f.ID=e.FractionationID) ";
$sql.="INNER JOIN Separations AS s on s.ID=f.SeparationID) ";
$sql.="LEFT JOIN Access AS a ON a.RowID=s.ID) ";
$sql.="LEFT JOIN Members AS m ON a.GroupID=m.GroupID) ";
$sql.="LEFT JOIN Groups AS g ON g.ID=m.GroupID ";
$sql.="WHERE ((m.UserID='$uid' AND a.Table='Separations') ";
$sql.="OR s.OperatorID='$uid' OR g.Name='Guests') AND UPPER(Spectrum) LIKE UPPER('%$query%')";
//echo"$sql  
";
mysql\_query($sql);
$rs = GetResultTable("DISTINCT dta,pepID","identi","");
if($rs) {
$results = count($rs);
echo"";
echo"\n";
echo"Found $results **Identifications(s)** that match your query. ";
if($results>$limit)
echo" Show all  
\n";
else
echo"  
";
echo"";
echo"

\n";
$sLim = $limit;
if($sLim > $results)
$sLim = $results;
echo"

";
for($o=0; $o<$sLim; $o++) {
$row = $rs[$o];
echo"|  |
| --- |
| $row[0] |
\n";
}
echo"

  
\n";
}
///////////////////////////////////////////////////////////////////
$rs = GetResultTable("\*","Organisms","UPPER(Genus) LIKE UPPER('%$query%') OR UPPER(Species) LIKE UPPER('%$query%') OR UPPER(Strain) LIKE UPPER('%$query%') OR UPPER(Description) LIKE UPPER('%$query%')");
if($rs) {
$results = count($rs);
echo"";
echo"\n";
echo"Found $results **Organism(s)** that match your query. ";
if($results>$limit)
echo" Show all  
\n";
else
echo"  
";
echo"";
echo"

\n";
echo"

\n";
$sLim = $limit;
if($sLim > $results)
$sLim = $results;
for($o=0; $o<$sLim; $o++) {
$row = $rs[$o];
echo"|  |  |  |
| --- | --- | --- |
| *$row[1]* | *$row[2]* | $row[3] |
";
}
echo"

\n";
echo"

  
\n";
}
///////////////////////////////////////////////////////////////////
//mysql\_close();
} else {
?>
**The following queries are supported:**  
• Transcript Numbers  
• Protein Sequences (min. 4 Characters)  
• Peptide Sequences (min. 4 Characters)  
• Experiment Names  
• Spot Names  
• Organisms  
php
}
?

  
php include("layout/footer.php"); ?
