# Supplementary material for: 2DB: a Proteomics database for storage, analysis, presentation, and retrieval of information from mass spectrometric experiments
Source: BMC Bioinformatics. 2008 Jul 7;9:302. doi: 10.1186/1471-2105-9-302 (PMC2475538; doi:10.1186/1471-2105-9-302)
Supplement: Additional file 1 — All files needed to run and further develop the database application as well as the user manual have been bundled into one zip file which can be downloaded from biomedcentral here. Due to constant upgrading of the system, it may be beneficial to check for the latest version on our website [12]. All the sources and additional installation files. [file 1471-2105-9-302-S1.zip › admin/pool.php]

2DB - Pool Experiments
php
include("../layout/menu\_admin.php");
//////////////////////////////////////////////////
// //
// Search Results... //
// //
//////////////////////////////////////////////////
echo "<bPool Experiments:  
  
";
if (!isset ($\_COOKIE["login"])){
header ("Location: ../index.php");
}
else{
echo "\n";
echo "

\n";
echo "|  |  |
| --- | --- |
|\n";
echo " **Select Experiments:** |\n";
echo " [?] |\n";
echo "
\n";
echo "|\n";
echo "  |\n";
echo "
\n";
echo "|\n";
echo " \n"; echo "\n"; $rs = GetResultTableSQL("SELECT ID, Name, Date FROM Separations"); if(!$rs){ echo"----------\n"; }else{ for($i=0; $i$row[1] ($datum)\n"; } } echo "\n"; echo " |\n";
echo " \n"; echo "\n"; if(!$rs){ echo"----------\n"; }else{ $rs = GetResultTableSQL("SELECT ID, Name, Date FROM Separations"); for($i=0; $i$row[1] ($datum)\n"; } } echo "\n"; echo " |\n";
echo "|\n";
echo "

  
\n";
echo "\n";
echo "\n";
echo "  
\n";
echo "  
\n";
if($select == "select"){
if($experiment1 == $experiment2){
echo "You cannot pool data from the same experiment!  
";
}else{
echo "\n";
echo "

\n";
echo "|  |  |  |
| --- | --- | --- |
|\n";
echo " Experiment: $experiment1 |\n";
echo " Pool |\n";
echo " Experiment: $experiment2 |\n";
echo "
\n";
echo "|\n";
echo "  |\n";
echo "
\n";
echo "|\n";
$rs = GetResultTableSQL("SELECT ID, Name FROM Fractionations WHERE SeparationID = '$experiment1' ORDER BY Name");
for($i=0; $i\n";
echo " "; echo "\n"; echo " |\n";
echo " ------- |\n";
echo " "; echo "\n"; echo"----\n"; $rss = GetResultTableSQL("SELECT ID, Name FROM Fractionations WHERE SeparationID = '$experiment2' ORDER BY Name"); for($j=0; $j$rows[1]\n"; } echo "\n"; echo " |\n";
echo "
\n";
}
echo "\n";
echo "

\n";
echo "  
\n";
echo "  
\n";
}
echo "\n";
echo "\n";
}
if($pool == "pool data"){
echo "Pool Is COOL!  
";
}
}
?>
php include("../layout/footer.php"); ?
