# Supplementary material for: 2DB: a Proteomics database for storage, analysis, presentation, and retrieval of information from mass spectrometric experiments
Source: BMC Bioinformatics. 2008 Jul 7;9:302. doi: 10.1186/1471-2105-9-302 (PMC2475538; doi:10.1186/1471-2105-9-302)
Supplement: Additional file 1 — All files needed to run and further develop the database application as well as the user manual have been bundled into one zip file which can be downloaded from biomedcentral here. Due to constant upgrading of the system, it may be beneficial to check for the latest version on our website [12]. All the sources and additional installation files. [file 1471-2105-9-302-S1.zip › install/Install.php]

Installation Step1


## Installation

**Step**  
**1**  
**⇒**  
**2**  
**⇒**  
**3**  
**⇒**  
**4**

### Checking Server and Browser requirements:

Before you can install 2DB, your hardware and software requirements are tested.
If the minimum requirements are not fullfilled the database couldn´t be installed

  
 **Server requirements:**  
  
php
/////////////////////////////
///// PHP Version Check /////
/////////////////////////////
$PHPVersion = phpversion();
$phptmp = str\_replace(".", "",$PHPVersion);
if($phptmp = "439"){
$color = "green";
$php = "true";
}
else{
$color = "red";
$error = "- Minimum: 4.3.9";
}
echo "

\n";
echo"Your PHP-Version: $PHPVersion $error";
echo "

  
";
///////////////////////////////
///// MYSQL Version Check /////
///////////////////////////////
$MYSQLVersion = mysql\_get\_client\_info();
$mysqltmp = str\_replace(".", "",$MYSQLVersion);
if($mysqltmp >= "4120"){
$color = "green";
$mysql = "true";
}
else{
$color = "red";
$error = "- Minimum: 4.1.20";
}
echo "

\n";
echo"Your MySQL-Version: $MYSQLVersion $error";
echo "

  
";
//////////////////////////
///// Download Check /////
//////////////////////////
function ConvertFromIniValue($value,&$type) {
$value = str\_replace("TB","T",$value);
$value = str\_replace("Tb","T",$value);
$value = str\_replace("tB","T",$value);
$value = str\_replace("tb","T",$value);
$value = str\_replace("t","T",$value);
$value = str\_replace("GB","G",$value);
$value = str\_replace("Gb","G",$value);
$value = str\_replace("gB","G",$value);
$value = str\_replace("gb","G",$value);
$value = str\_replace("g","G",$value);
$value = str\_replace("MB","M",$value);
$value = str\_replace("Mb","M",$value);
$value = str\_replace("mB","M",$value);
$value = str\_replace("mb","M",$value);
$value = str\_replace("m","M",$value);
$value = str\_replace("KB","K",$value);
$value = str\_replace("kb","K",$value);
$value = str\_replace("Kb","K",$value);
$value = str\_replace("kB","K",$value);
$value = str\_replace("k","K",$value);
$value = str\_replace("Bytes","B",$value);
$value = str\_replace("bytes","B",$value);
$val = substr($value,0,strlen($value)-1);
$type = substr($value,strlen($value)-1);
switch($type) {
case 'T' : $val \*= 1024; $type="K";
case 'G' : $val \*= 1024; $type="G";
case 'M' : $val \*= 1024; $type="K";
case 'K' : $val \*= 1024; $type="B"; break;
default : $val = $value; $type="";
}
return($val);
}
function ConvertToIniValue($value,$type) {
$newValue = $value;
while(1) {
$newValue = $value / 1024;
if($newValue > 1) {
$value = round($newValue,0);
increaseType($type);
} else
break;
}
return("$value$type");
}
function increaseType(&$type) {
switch($type) {
case 'B' : $type='K'; break;
case 'K' : $type='M'; break;
case 'M' : $type='G'; break;
case 'G' : $type='T'; break;
default : $type='X';
}
}
function PHPINIWertUmwandeln($Groesse,&$type)
{
$Werte['MB'] = 1048576;
$Werte['Mb'] = 1048576;
$Werte['M'] = 1048576;
$Werte['m'] = 1048576;
$Werte['KB'] = 1024;
$Werte['Kb'] = 1024;
$Werte['K'] = 1024;
$Werte['k'] = 1024;
while(list($Schluessel)=each($Werte))
{
if((strlen($Groesse)>strlen($Schluessel)) && (substr($Groesse, strlen($Groesse)-strlen($Schluessel))==$Schluessel))
{
$Groesse=substr($Groesse, 0, strlen($Groesse)-strlen($Schluessel))\*$Werte[$Schluessel];
$type = $Werte[0];
break;
}
}
return $Groesse;
}
function UploadmoeglichkeitPruefen()
{
$Uploadmoeglichkeit=TRUE;
if(strtolower(@ini\_get('file\_uploads'))=='off' || @ini\_get('file\_uploads')==0)
{
$Uploadmoeglichkeit=FALSE;
}
return $Uploadmoeglichkeit;
}
function MaximaleDateiuploadGroesseErmitteln()
{
$MaximaleDateiuploadGroesse=0;
if($Dateigroesse=ini\_get('upload\_max\_filesize'))
{
$MaximaleDateiuploadGroesse=PHPINIWertUmwandeln($Dateigroesse,$type);
}
if($Postgroesse=ini\_get('post\_max\_size'))
{
$Postgroesse=PHPINIWertUmwandeln($Postgroesse,$type);
if($Postgroesse<$MaximaleDateiuploadGroesse)
{
$MaximaleDateiuploadGroesse=$Postgroesse;
}
}
return$MaximaleDateiuploadGroesse;
}
$met = ConvertFromIniValue(ini\_get('max\_execution\_time'),$type);
$nmet = $met+1;
ini\_set('max\_execution\_time', $nmet.$type);
$smet = ini\_get('max\_execution\_time');
if($nmet == $smet) {
echo "

\n";
echo"The max execution time for scripts can be automatically adjusted.  
";
echo"It is currently $met s  
";
echo "

  
";
ini\_set('max\_execution\_time', $met);
} else {
echo "

\n";
echo"The max execution time cannot be automatically adjusted.  
";
echo"It is currently $met s  
";
echo "

  
";
}
////////////////////////////////////////////////////////////////
///// Max Upload
////////////////////////////////////////////////////////////////
$met = ConvertFromIniValue(ini\_get('upload\_max\_filesize'),$type);
$oval = ConvertToIniValue($met,$type);
$nmet = $met+1024\*1024;
$ival = ConvertToIniValue($nmet,$type);
ini\_set('upload\_max\_filesize', $ival);
$test = ConvertFromIniValue(ini\_get('upload\_max\_filesize'),$type);
$nval = ConvertToIniValue($test,$type);
//echo"O: $oval, I: $ival, N: $nval  
";
if($ival == $nval) {
echo "

\n";
echo"The max upload property restricts the file size that can be uploaded to the server.  
";
echo"This affects your ability to upload sequences and measurements as well as images.  
";
echo"For sequences or data you may split one large into multiple smaller files in the worst case.  
  
";
echo "The max upload size for scripts can be automatically adjusted.  
";
echo"It is currently $oval  
";
ini\_set('upload\_max\_filesize', $oval);
} else {
echo "

\n";
echo"The max upload property restricts the file size that can be uploaded to the server.  
";
echo"This affects your ability to upload sequences and measurements as well as images.  
";
echo"For sequences or data you may split one large into multiple smaller files in the worst case.  
  
";
echo"The max upload file size cannot be automatically adjusted.  
";
echo"It is currently $oval  
  
";
echo"In order to increase this value, you need to contact your ISP.  
";
}
echo "

  
";
////////////////////////////////////////////////////////////////
///// Max Post
////////////////////////////////////////////////////////////////
$met = ConvertFromIniValue(ini\_get('post\_max\_size'),$type);
$oval = ConvertToIniValue($met,$type);
$nmet = $met+1024\*1024;
$ival = ConvertToIniValue($nmet,$type);
ini\_set('post\_max\_size', $ival);
$test = ConvertFromIniValue(ini\_get('post\_max\_size'),$type);
$nval = ConvertToIniValue($test,$type);
//echo"O: $oval, I: $ival, N: $nval  
";
if($ival == $nval) {
echo "

\n";
echo "This option affects the max size of post data allowed. This setting also affects file upload. To upload large files, this value must be larger than upload\_max\_filesize.  
  
";
echo "The max upload size for scripts can be automatically adjusted.  
";
echo"It is currently $oval  
";
ini\_set('post\_max\_size', $oval);
} else {
echo "

\n";
echo "This option affects the max size of post data allowed. This setting also affects file upload. To upload large files, this value must be larger than upload\_max\_filesize.  
  
";
echo"The max upload file size cannot be automatically adjusted.  
";
echo"It is currently $oval  
  
";
echo"In order to increase this value, you need to contact your ISP.  
";
}
echo "

  
";
////////////////////////////////////////////////////////////////
///// Max memory
////////////////////////////////////////////////////////////////
$met = ConvertFromIniValue(ini\_get('memory\_limit'),$type);
$oval = ConvertToIniValue($met,$type);
$nmet = $met+1024\*1024;
$ival = ConvertToIniValue($nmet,$type);
ini\_set('memory\_limit', $ival);
$test = ConvertFromIniValue(ini\_get('memory\_limit'),$type);
$nval = ConvertToIniValue($test,$type);
//echo"OO: $met, O: $oval, I: $ival, N: $nval  
";
if($ival == $nval) {
echo "

\n";
echo "This sets the maximum amount of memory in bytes that a script is allowed to allocate. This helps prevent poorly written scripts for eating up all available memory on a server. It may impair the upload of new data to the database if it is too low (less than 100 MB).  
  
";
echo "The max memory size for scripts can be automatically adjusted.  
";
echo"It is currently $oval  
";
ini\_set('memory\_limit', $oval);
} else {
echo "

\n";
echo "This sets the maximum amount of memory in bytes that a script is allowed to allocate. This helps prevent poorly written scripts for eating up all available memory on a server. It may impair the upload of new data to the database if it is too low (less than 100 MB).  
  
";
echo"The max memory size cannot be automatically adjusted.  
";
echo"It is currently $oval  
  
";
echo"In order to increase this value, you need to contact your ISP.  
";
}
echo "

  
";
/\*
Obsolete since spectrum is now displayed in java applet
if (!extension\_loaded('gd')) {
if (!dl('gd.so')) {
echo "

\n";
echo"The gd module is not enabled on your server.  
";
echo"This will prevent you from using hotspots on images.  
";
echo"The spectra will also not be displayed.  
";
echo "

  
";
}
} else {
echo "

\n";
echo"GD module is installed  
";
///Theoretical spectrum
$content = "";
$rs = popen("../spectra/NewFrag WLQYSEVIHAR test.dta 1","r");
while(!feof($rs)) {
$line = fread($rs,8192);
if($line == "")
break;
$content .= $line;
}
pclose($rs);
$len = strlen($content);
settype($len,"integer");
if($len < 10) {
echo"You cannot see the theoretical spectrum, though.  
";
} else {
echo"You can see the theoretical spectrum as well.  
";
}
echo "

  
";
}
\*/
if(UploadmoeglichkeitPruefen())
{
$MaximaleDateiuploadGroesse=MaximaleDateiuploadGroesseErmitteln();
$upload = "true";
echo "

\n";
echo"You are allowed to upload files!";
echo "

  
";
echo "

\n";
echo "Maximum file size: ",
number\_format($MaximaleDateiuploadGroesse/1024/1024, 0, ",", "."),
" MB (",
$MaximaleDateiuploadGroesse,
" Bytes)  
";
echo"Files you can upload such as sequences may not exeed this limit.";
echo "

  
";
}
else
{
echo "

\n";
echo"You are not allowed to upload files!";
echo "

  
";
}
?>
 **Browser requirements:**  
  


Please activate JavaScript

  


Please activate Java

  


Please activate Cookies

 
