# Supplementary material for: 2DB: a Proteomics database for storage, analysis, presentation, and retrieval of information from mass spectrometric experiments
Source: BMC Bioinformatics. 2008 Jul 7;9:302. doi: 10.1186/1471-2105-9-302 (PMC2475538; doi:10.1186/1471-2105-9-302)
Supplement: Additional file 1 — All files needed to run and further develop the database application as well as the user manual have been bundled into one zip file which can be downloaded from biomedcentral here. Due to constant upgrading of the system, it may be beneficial to check for the latest version on our website [12]. All the sources and additional installation files. [file 1471-2105-9-302-S1.zip › install/upgrade.php]

2DB - Download
php
extract($HTTP\_GET\_VARS);
extract($HTTP\_POST\_VARS);
@include("../functions.php");
@include("../Connect.php");
$tab = GetCookie("login");
$db = ConDB($dbname);
if(!$db) {
?
php
die("Couldn't connect to local database <br");
}
?>
**Upgrade**  
  
php
$rs = GetResultTableSQL("SELECT Name FROM Members INNER JOIN Groups ON Members.GroupID = Groups.ID WHERE UserID='$tab[1]' AND Name = 'Administrators'");
if(!$rs) {
?
php
die("You need to be logged in as an administrator!");
}
settype($locVersion,"double");
$h = @fopen("$link/SchemaUpdates.sql","r");
if($h) {
$sqlUpgrade = "";
while (!feof($h)) {
$sqlUpgrade .= fgets($h, 4096);
}
pclose($h);
$versionArr = split(">>>",$sqlUpgrade);
array\_shift($versionArr); //Get rid of the first row (contains no information)
for($i=0; $i $locVersion) {
$statement = "";
foreach($farr as $line) {
$statement .= $line . " ";
if(strpos($line,";") !== false) {
$statement = str\_replace("\n"," ",$statement);
$statement = str\_replace("\r"," ",$statement);
$statement = str\_replace(";","",$statement);
$rs = @mysql\_query($statement);
$statement = "";
}
}
}
}
echo"Successfully upgraded schema information.  
";
} else
echo"Couldn't connect to schema upgrade service.  
";
$fileUpgrade = @fopen("$link/FileUpdates.txt",'r');
if($fileUpgrade) {
$content = "";
while (!feof($fileUpgrade)) {
$content .= fgets($fileUpgrade, 4096);
}
$filesArr = split("////File////",$content);
array\_shift($filesArr); //Get rid of the first row (contains no information)
foreach($filesArr as $file) {
$farr = split("\n",$file);
$tmp = "";
do {
$tmp = array\_shift($farr);
} while(strlen($tmp) < 3 && count($farr) > 1);
list($relLoc,$name,$access,$secVersion) = split(" ",rtrim(ltrim($tmp)));
settype($secVersion,"double");
if($secVersion > $locVersion) {
$filePath = "$relLoc/$name";
if(@file\_exists($filePath))
@unlink($filePath);
$handle = @fopen($filePath, 'w');
if($handle) {
foreach($farr as $line) {
fwrite($handle, $line);
}
fclose($handle);
//@chmod("$filePath",$access);
} else
echo"Couldn't write local file ($relLoc,$name).  
";
}
}
echo"Successfully upgraded GUI implementation.  
";
} else
echo"Couldn't connect to file upgrade service.  
";
?>
  
  
