# Supplementary material for: 2DB: a Proteomics database for storage, analysis, presentation, and retrieval of information from mass spectrometric experiments
Source: BMC Bioinformatics. 2008 Jul 7;9:302. doi: 10.1186/1471-2105-9-302 (PMC2475538; doi:10.1186/1471-2105-9-302)
Supplement: Additional file 2 — All files needed to run and further develop the database application as well as the user manual have been bundled into one zip file which can be downloaded from biomedcentral here. Due to constant upgrading of the system, it may be beneficial to check for the latest version on our website [12]. The user manual for both installation and usage. [file 1471-2105-9-302-S2.doc]

# Home

Welcome to 2DB.

This web-based database system allows storage, analysis, and displays of experimental data from, for example, 2 dimensional gel electrophoresis and mass spectrometry.

Anything that can be presented as an image can be displayed in this database. Areas e.g. hot spots on such an image can be selected. The data gained from mass spectrometric analysis of these spots can also be stored (software independent e.g. from Sequest, Mascot, ..).

The aim, protein identification, can easily be achieved with this tool. Set your thresholds once and whenever a spot or a gel is accessed, the proteins are detected on the fly in respect to the thresholds.

This version of the database is based on the sequence of Chlamydomonas reinhardtii. It displays all published information to anyone, but also contains our current projects, which are however not displayed to the outside.

If you have access to a web-server allowing PHP and MySQL, feel free to download our extremely easy installation package. You can find the latest installation version at http://www.2db.de.ms.

# Download

You can only download the most current version of this database. While we are continuously developing, updates may become available. In case you would like to update, simply load the most current version and install it. Data will not be lost in this process, only new functionality will be gained. Some issues cannot be resolved in this fashion, which is why we included an update facility into the software.

## Installation

The installation is fairly easy and only requires minimal knowledge. No programming is required at any point.

## What do I need?

A web-server, whether locally installed or somewhere on the internet, is up to you. However, locally administering a web-server is a difficult task today and we suggest a professional package.

This web-server needs to provide CGI, PHP, and a MySQL database (older versions are mostly fine). Some functions require actual executables to be run, but they are not essential. Some web-space is required, too. A GB might be a good idea, but it depends on the amount of data you are keeping in the database (20 medium size experiments may amount to 50 MB.

In addition you need to know how to upload files to your web-space.

You will also need all the login information to your database.

## Getting Started

Equipped with the information above, simply unzip the downloaded file and upload the directories and files to your web-server. After that run the install.php file. Do this online. There you will be asked all the necessary data as indicated above. In addition to that, you have to set-up the first administrator.

The install.php file will indicate other measures you have to take to have a fully functional database (For instance protein sequences, gene models, or the like are required).

# Help

# Installing 2DB

### Pre Install

The installation of 2DB is dependent on the software environment. The requirements that need to be fulfilled in this regards are listed below. When choosing a web space make sure that this check list is fulfilled.

- A MySQL database of a version 4.1 or later needs to be available
- PHP version 4.0 or late needs to be available
- The server needs to be LINUX or UNIX
- Scripts including executables need to be allowed
  - Mass spectra may not be displayed if executables are not supported or if the server’s operating system is not UNIX/ LINUX
- You need to have ftp access.
- Upload of files needs to be allowed (the larger the better).

Once you decided on a server you may run our installation test script which will test whether 2DB will be able to run successfully in your environment.

Before running the installation script you need to gather some information. This information is listed below. Generally this information will be provided by your web space provider (ISP).

- You need to now the login information for your MySQL database
  - The location of the server
  - Your username
  - Your password
  - The name of the database

### Installation

Equipped with this information you can start the next step which is uploading 2DB to your web space. This can be done in a few easy steps.

1. Download the 2DB zip file
2. Extract the zip file to a folder on your computer
3. Upload all files and folders that were extracted to a directory on your web server.
   1. You will later connect to the database using that directory name e.g.: http://www.myWebSpace.com/theDirName

Now you are ready to install 2DB. All you have to do is type in the address of the location of the installation script of 2DB. This is somewhat dependent on your upload directory so the following is just an example:

http://www.myWebSpace.com/theDirName/install/install.php

After successfully starting the install.php script for the first time, you will be asked to enter the information you gathered above. If a successful connection to the database can be established you are than asked to enter some personal information which identifies you as the first administrator of the database. You may add further users and administrators later.

At this point the installation is done and the database can be used.

There are however a number of steps you may want to perform to customize your database. The post installation steps are described below.

### Post Installation

If you plan to be the sole user of the database, you do not need to enter more user information. However, if there will be multiple users that shall receive the right to upload experimental data to the database, you need to add all these users to the database before they can connect and use it. This procedure is described in the administration section of this manual under the section Add User.

This database helps you to map your identifications to protein sequences in the database. The prerequisite for this is that these sequences need to be present in the database. Therefore you need to upload some sequences to the database in order to be able to use this feature. This procedure is again described in the administration section of this manual under the heading Sequence Upload.

A small checklist may help to not overlook some of the initialization steps necessary to successfully work with the database.

- Add groups ()
- Add users ()
- Add sequences ()
- Add software and thresholds ()
- Add organism information ()

After these steps you are completely done and can now upload experimental information to the database in order to make full use of it.

## General

### Login


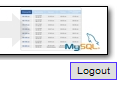

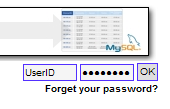
The user login is located on the upper right hand corner of the display area in the browser. There are two fields, which are filled with the information “UserID” and a sample password. Change the value “UserID” to your email address. Then enter your password in the input box right of it and press OK. If your login was successful, the login area will disappear and will be replaced by a single logout button. You will also be greeted by name in the text on the site.

### Forgot the Password

In case you forgot your password you can click on the link “Forgot your password?” right underneath the login area. Since your password is not stored in text form in the database, there is no way to retrieve it if you forget about it. Therefore you will be asked to enter your email address (Figure 1). A new password will be send to the specified email address if this email is available in the database.


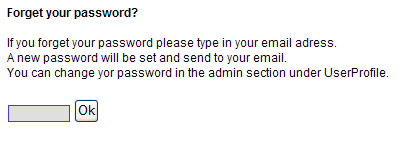


Figure 1: This screen will appear if you follow the hyperlink “Forgot your password?”. Here you enter your password and a new temporary password will be send to you.

Retrieve your mails from that account and login with the new password. You can than change your password again in the administration area under User-Profile.

### Change your Password


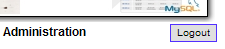

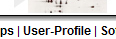
Once you are successfully logged on to the database i.e. the login area transformed into a logout button, you may enter the administration area to change your password. First click on the administration link to enter the administration area. In that area you will find the link User-Profile. Enter the user profile to change any data that is in regards to you.
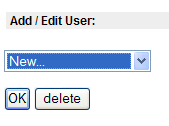
In order to change anything you need to first select your user name from the drop down box. Once you selected the correct user name you may press OK in order to review your settings. Only administrators are able to delete users or to enter new users to this list. So ignore these options in case you are not an administrator for the current instance of the 2DB database. After you pressed OK all information on you can be changed in the following dialog (Figure 2). The most important option here is to change your password and email address. This may be necessary occasionally when you change your email address, or when you happen to forget your password and you want to change the automatic password to some, for you, more meaningful value. Change the information you need to change and press the Update button. If you want to change your password, you need to first enter your current password (old) and then enter whichever password you would like to change the current one to (new). We have faith in you and are hopeful that we don’t have to force you to enter the new password twice and check the equivalence of the new password to itself as is done on many other pages.

This means that you need to make sure, that whatever you enter as your new password will be exactly what you intend to write there. Otherwise you need to use “Forgot the Password?” link again and redo the procedure associated with that.


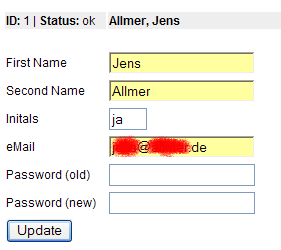


Figure 2: User data that can be changed in the User-Profile page of 2DB.

Another important part in this area is that you are able to set and review your thresholds for import of data from mass spectrometric and computational processing (Figure 3).


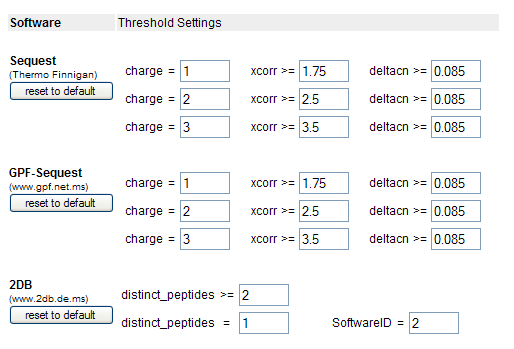


Figure 3: Threshold settings for some software that may be used in the database. Settings for 2DB will always be present.

Any software and its possible thresholds seen in this area needs to be entered by an administrator in the software area before. Once it has been established, users can change their thresholds which may be important for some reasons. The above is only an example of how this may look, but you will probably used different software than seen here. The last item 2DB will however always show up in any instance of the 2DB database.

The last item in the User-Profile shows which groups you are part of and this can onşy be adjusted by an administrator. So if you need to be part of some group let your admin know and you can be entered to that group.

# Administration

The administration are of 2DB requires a valid login. Anyone that has a valid login and is not just a guest user may enter the administration area. However, the actions one may perform depend on the rights set for the group that one is a member of. Therefore, it can be restricted whether a user is able to upload experimental data or if s/he should be allowed to perform various other operations.

If you are eligible to enter the administration area you will receive some information upon successful opening of the page. Your group memberships will be displayed and some information about current and maximum size of the database are displayed (Figure 4).


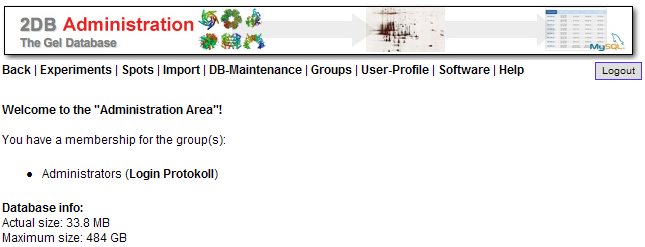


Figure 4: Administration welcome screen.


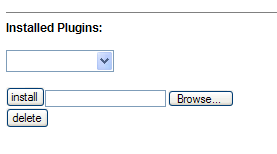


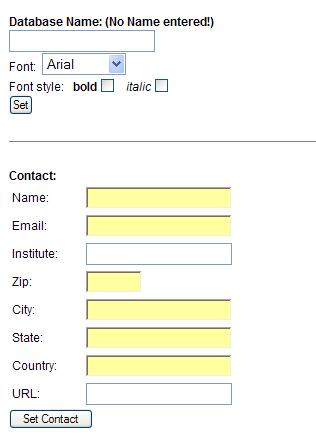
This welcoming screen may look different in your instance of 2DB, but it only displays some general information. The information that needs attention of administrators in this area is accessible via the hyperlinks at the top which have changed due to successful opening of the administration area. In order to leave the administration area again you need to follow the Back hyperlink on the left side. Some information about your instance of 2DB can be customized here however.

You may want to use customized plugins for view of sequences in the database. Here is the place to upload and delete plugins to make them available for the users.

Since you would probably like to set the name of your database which can be done in the first area (see right). Then your contact information needs to be available on the webpage. Some information can be entered as seen to the right. Feel free to enter as much or little information as you like. Use the button set to set the name of the database and the button Set Contact to update your contact information.

### Experiments


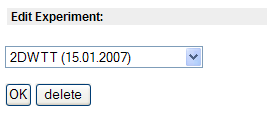
This section can be entered by users from some groups and administrators. Here the information to a specific experiment in the database can be inserted, deleted, or updated. From the first drop-down box in this section you may choose one of the experiments that have been entered into the database or select new to enter new information (Figure 5). Upon successful selection, a form as seen in Figure 5 will be available.


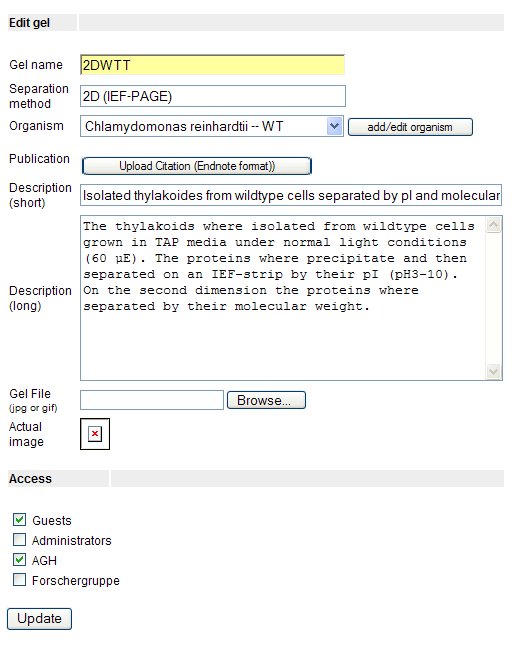


Figure 5: View of the experiment section from the administration area.

The information concerning your experiment can be set here. First you need to enter a name for the experiment (misleadingly labeled gel). Than you can enter more information such as the methods used, some further description and so forth. In order to upload a picture which shall represent your experiment use the Browse button? Here no picture has been set. Otherwise it would show up instead or the place holder with the red cross. Once the data has been entered a button which allows the uploading of references for this experiment can be accessed. You may upload multiple citations for each experiment. They will then be available as DOI links in the preview and the detailed view of the experiment.

One crucially important area here is the Access area which allows for the setting of groups that are allowed access to the information of this experiment. If you allow Guests to access this information than anyone that may find the website can access the information. We usually allow this access upon publishing the information in a journal.

### Spots


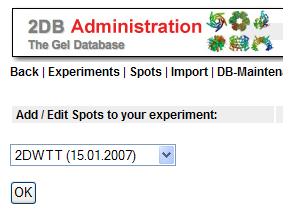
This section of the administration area enables the definition of areas on a graphic representation of the experiment. For 2D gels or anything that can be displayed as a picture with definite areas of interest on the image. This means that the individual spots may be selected and connected to data in the database. The procedure is extremely easy, requires however a working installation of a Java virtual machine. Since this is usually the case there is nothing in the way of connecting data and hot spots on the image.

First you need to select the experiment you want to work with. At this point you should also be aware of the fact that a picture must have been uploaded prior to using this utility. Pictures can be uploaded in the Experiments section.

Just select an experiment from the drop-down menu and press OK. Now a Java window will open and after some time the graphical representation for this experiment will be displayed in the Java window.

In the list on the left side of the window all available spots are listed. Choose any of these spots by merely clicking on the entry in the list. Now you can link to an area on the picture by simply clicking and dragging to create either a circular or rectangular shape. This shape will later define the clickable area for that image. When the area is later clicked all available information such as identified peptides and proteins as well as their supporting information will be displayed.

The Java applet (Figure 6) is generally divided into four sections. On the left there is a list of available spots from the database. These spots can be selected, as is the spot named “B3-2” in this case. Below that list is the information which is currently known for that entry. Here only name and coordinates are known. The value “-1” means unknown value in this case. If the selected entry has coordinates specified the outline of these coordinates will be highlighted in red in the actual picture frame. Above the picture, the zoom level and the form, either circle or rectangle, can be chosen. Use these forms to define a clickable area to be connected with any name in the list retrieved from the database. You may perform this in as many sessions as you like until you marked all areas that you processed from the “gel”.


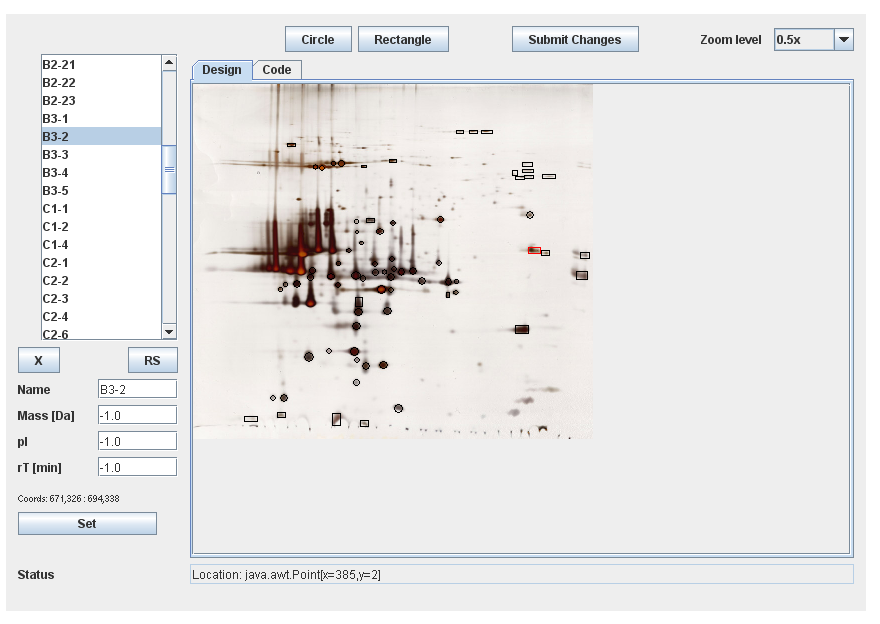


Figure 6: JAVA applet enabling a user friendly definition of areas on a picture (here 2D PAGE).

Details on how the areas can be defined practically are found below.

#### Instructions

Hopefully the applet is intuitive enough to be used without instructions! Nonetheless, here are some hints, tips, and tricks:

##### Adding an Area

Select some point in the image, press the left mouse button, drag the mouse to the desired position, and release the left mouse button again. Did you notice the green frame that showed you the Area which will be clickable in the browser when the image map is one? After releasing the left mouse button the outline of the area turns black and in the left-hand list the coordinates of the area shows up.

##### Adjusting the area

Once an area has been defined it can still be adjusted which might be necessary if you didn't hit the exact spot. In order to start adjusting an area click it in the image area until the outline turns red. The thus selected area can now be modified with the following key commands:

| **l** | Moves the area to the left (5px) |
| --- | --- |
| **u** | Moves the area up (5px) |
| **r** | Moves the area to the left (5px) |
| **d** | Moves the area to down (5px) |
|  |  |
| **shift+l** | Moves the area to the left (1px) |
| **shift+u** | Moves the area up (1px) |
| **shift+r** | Moves the area to the left (1px) |
| **shift+d** | Moves the area to down (1px) |
|  |  |
| **alt+l** | Extends the right border to the right (5px) |
| **alt+u** | Extends the upper border to the top (5px) |
| **alt+r** | Extends the right border to the right (5px) |
| **alt+d** | Extends the lower border to the bottom (5px) |
|  |  |
| **shift+alt+l** | Extends the right border to the right (1px) |
| **shift+alt+u** | Extends the upper border to the top (1px) |
| **shift+alt+r** | Extends the right border to the right (1px) |
| **shift+alt+d** | Extends the lower border to the bottom (1px) |
|  |  |
| **v** | Shifts the top border towards the bottom border (5px) |
| **h** | Shifts the left border towards the right border (5px) |
|  |  |
| **shift+v** | Shifts the top border towards the bottom border (1px) |
| **shift+h** | Shifts the left border towards the right border (1px) |

Another way to adjusting the area is to select an area in the left-hand list and than simply create a new area as described above. The old area will be discarded and the new one will show up in the list.

##### Zoom level

Some images may be too big or too small to comfortably work with them. Therefore, you can choose a zoom level from the drop-down box in the upper right hand corner. If you loose your area while zooming, simply click on the entry in the list and the area will be brought into focus.

##### Shape

There are two buttons which let you choose in-between a circle as the area and a rectangle. Unfortunately, image maps do not support ovals. Press the buttons to switch in-between the two options.

##### Adding data

In order to construct a meaningful image map it is not enough to define the areas. To specify further information, select an area in the list and enter the extended information in the fields below the list.

**Name**
Here you can give your spot another name.

**Mass**
Here you can fill in the mass of your spot.

**pI**
Here you can fill in the pI of your spot.

**rT**
Here you can fill in the retention time for your spot.

##### Area List

On the left hand side is a list which displays all the areas currently associates with the image.

**Button X**
This button deletes the currently selected entry from the area list.

**Button RS**
This button deletes the coordinates from the currently selected entry in the area list.

### Add groups

*General:*

Users are not able to make any changes.

*For Administrators:*


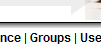
There are two special groups set-up during the installation of 2DB. One is the administrators group which allows all operations possible for 2DB. The other group is the guest group which only allows viewing of published data.

It may be of use to add at least one more group which will consists of all members of the research group and which should have the right to upload data. It might further be interesting to create a group for collaborators which may see information that is not yet available for the public.


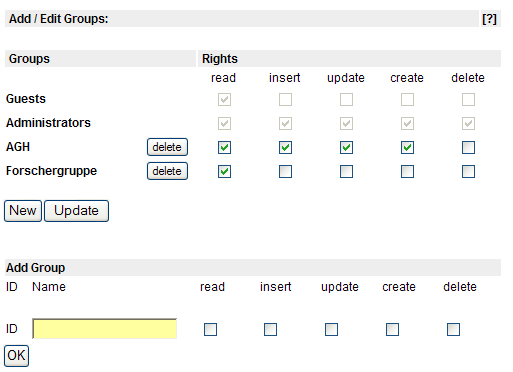


The access rights that need to be set during creation of new groups are described below.

#### Access rights

All access rights that can be set for a group modify the ability of members of that group to perform certain operations in the database.

##### Read

##### Insert

##### Update

##### Create

##### Delete

### Add users

Users other than guest users need to be added individually to the database. While creating a new user some information needs to be specified for that user. The most important value is the email address, which will also serve as the login later. The user also needs to be included into one or multiple groups in order to be able to perform the tasks allowed for those groups.


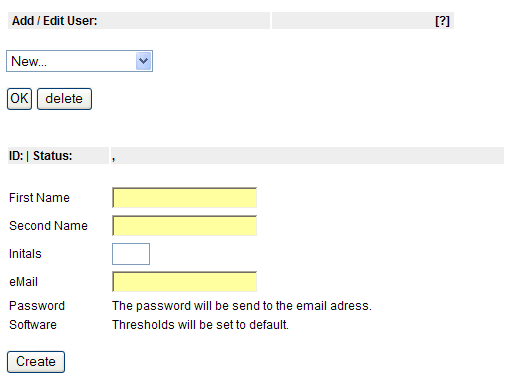


Once a user has been created s/he will get the password for 2DB mailed to the specified mail account. This password can be changed in the future by logging on to 2DB and than going to the administration area and then editing the account data in the User-Profile section.

#### Changing Passwords

Passwords can be changed by logging on to 2DB and than going to the administration area and then opening the User-Profile section.

Here both, old and new password, need to be specified in order to change the password. This procedure may be repeated whenever the password needs to be changed.

For details turn to the Login section.

#### Forgot Password

This problem may be resolved quite easily. Simply click on the link below the login area and in the page that opens specify your email address. If you have an account for 2DB a new password will be mailed to that account and you can than again change your password as described above.

2DB does only record your passwords encrypted and it is not possible to reverse engineer the encryption to retrieve your password. Therefore, 2DB needs to send you a randomly generated password in case you forgot your old one.

For details turn to the Login section.

### Adding Protein Sequences


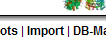
You need to be logged in as an administrator to be able to add protein sequences. This feature is located in the Administration area in the Import section.

This process is not trivial and needs special attention. So read the following information carefully and adhere to it exactly. Some things need to be performed before trying to upload the sequences (Pre Upload) and some operations need to be performed during upload (Uploading).

#### Pre Upload

The sequences you want to upload to the database need to be of FASTA format (see below). It would also be helpful to examine the header within the file to see whether it can be used to connect the protein information to the database it originates from.

This would make it possible to access further information on the identifications in your dataset by a single click. The process of how to connect information in the FASTA file to, for example entries in JGI or NCBI, will be described in detail below.

#### Protein Database Link

Open the file containing the protein sequences you would like to use within 2DB. Also open a web browser and connect to the database where you originally downloaded the sequences. The web site should have a search method which you should use to enter the information found in the first line of the sequence file. Try subsets of the information until you are able to retrieve the same protein as specified in the sequence file.

Now you can browse the web site and choose a page that you would like to link to the protein sequence. That means that a page displaying this information would open once you click on an identified protein in your dataset. A restriction is that it needs to be possible to retrieve this page with the information contained in the header and maybe some additional constants that are equal for all sequences in the sequence file.

The link that you followed to retrieve the detailed information for the protein you are looking at is usually displayed in the address bar on top of the browser. Copy this link and examine it in detail.

First extract the information prior to the question mark. This part of the link needs to be entered into the link field during uploading the file. This part was fairly easy and now all that is left to do is to examine the part of the address following the question mark.

Variables and values are separated by the equal sign and multiple pairs of these are separated by ampersand characters (&). Take each pair at a time and try to decide whether it is a variable or a constant. A constant would always be the same for the database and sequence file in question. This can be learned by entering the information from subsequent proteins in the sequence file instead of the information currently contained in the link. If it is possible to leave some name=value pair unchanged and still reach the correct protein information, it is probably a constant for this sequence file database combination.

On the other hand variables are dependent on the header information prior to the sequences in the sequence file. This information needs to be changed for each protein in order to retrieve its information.

Once you can successfully reach a page with detailed information for the proteins in the database based on constants and variables (dependent on the information in the header of the sequence file), you can proceed to actually uploading the sequence information.

### Uploading

Uploading data to your server may be restricted by your web space provider. The script will try to increase the limit as much as possible, but you may have to split the file into multiple smaller portions to perform the upload or notify your provider to increase the upload limit.

The script will ask for the location of the sequence file and if known to you for the separating character in the header. You should have noticed this character separating multiple values during examining the pre upload instructions. If there is none enter “-1”. If you don’t know, leave this field blank and proceed. Also make sure to enter the type of sequence you are uploading (gene models, protein sequences, or translated EST, …).

If you left the separator field blank, the header information of the first sequence is than displayed for you and you are asked to identify


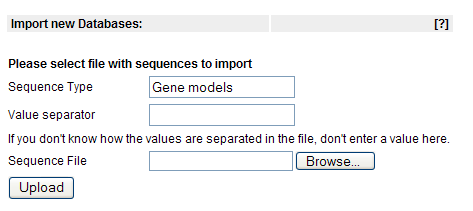


the separating character. Once this issue is resolved the next step will display the header split up into its components. Each of the components will be labeled by a number that you shall use to identify the variables necessary to retrieve more information on the protein (see above).

You are now asked to enter additional information for the sequences in the file. First choose the component of the header that you would like to have displayed as the identifier for that protein. Just enter the associated number into the Protein id field. If there is additional information on the protein contained in the header, enter the associated number into the description field (Figure).


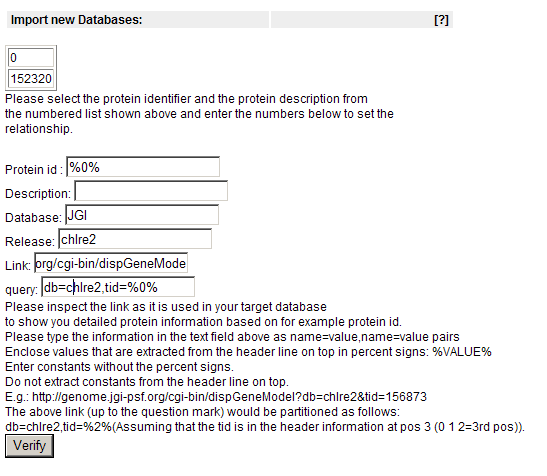


The fields database refer to information that you may need later to identify where this information comes from. Enter NCBI if you got your sequence files from there for example. The release is in reference to the sequence file. Here you should enter the release of the file in order to be able to resolve issues with different releases in the future.

Now comes the information you determined in the section pre upload. The link field takes the fraction prior of the question mark in the link. The query field takes the information following the question mark (don’t include it). Any escaped signs should be removed (usually start with % and are followed by a number e.g.:”%20”). Now all that is left are name=value&name=value pairs. The names should remain unchanged. The values may either be substituted by constants. In this case simply enter the constant value. They may also be substituted by variables from the header. In this case enter the position in the header enclosed in percent signs (“%0%” for the example in the figure above). Instead of the ampersand character commas are used. They need to be used to separate name=value,name=value pairs.

Once all the information is filled in, you can upload the information. During the time it takes to process and import the sequences into the database, information concerning the process is displayed continuously. Depending on the size and the speed of the database server, this may take a while.

### Import Restrictions

If a sequence is encountered multiple times in the sequence file, the information specified in the header is used as an alias. Only one link will however be created for all aliases.

If the same protein id is encountered multiple times during install, and the underlying sequences are different, the name of the protein is changed to:

Original name _ release _ running number (e.g.: ATP_rel.1.34_2).

Sequences are considered equal if all amino acids are the same and come in the same sequence.

Obviously, if protein id and sequence are the same for multiple entries in the sequence file, all subsequent instances are ignored.

If you keep failing to upload the file try a smaller one containing just one or two proteins. If that works, you should contact your web space provider in order to increase your upload limit.

### FASTA format

The format specifications are quite simple and loose. In my humble opinion it is hard to imaging how such a file type has become so popular.

A header for any subset of the database (e.g. a gene, or a protein sequence) is introduced by a greater than sign (>). Then the header follows for example:

>lhca3 gene product

This information may not exceed one line. No line breaks are allowed within the header. However, there may be further information included in the header. An example from JGI may show this:

>jgi|Chlre3|15|fgenesh1_pm.C_scaffold_8000015

The data presented on that line is not standardized and varies from each distributor of such files. It even varies within different JGI distributions of a sequence.

Then the actual information follows as either genetic information or protein information.

The genetic information must consist of the capitalized Letters ACGNT only. Note that numbers and white spaces are usually ignored so that depending on the tool in question they may be allowed in the text. Line breaks may be added as needed.

A fasta file would then look something like this:

>Gene or Scaffold 1

ACTGNNCGTNTNTNANANACCCGTCGCT

CGTCGTNANANACCACTGNNCGTNTNTT

>Protein 2

MKSDLFKWEORLKWLERKOIFLKSLDFI

SDFOEIWROEWNNDFMSDFMWELRHNFQ

Note that usually mixing of genomic and proteomic data is not supported as is done in the example above. Since the FASTA format is widely spread it is worth understanding it. Many web interfaces of commonly used tools in proteomic data analysis offer the input of several datasets in FASTA format.

### Adding Experimental Data

### Importing Data

Data can be imported by administrators
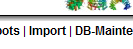
and users that have the right to insert. The location of the import facility is in the Administration area in the Import section.

The first section of that page allows the upload of data. There are multiple file formats that are allowed as input. They will be described below. No matter the file format, just use the Browse button to locate the file and once you selected the file it can be uploaded by clicking the Upload button. Some web space providers set a very low upload limit of 2MB. The script will try to increase the limit, but if that is not possible, you need to contact your provider to increase the limit.


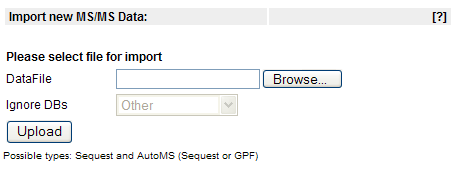


The file that you upload will be inspected and then processed line by line. Duplicate entries will be ignored. Duplicates are identifications by the same software for the same spectrum. If a given identification for a spectrum has a higher score than some other, the one with the higher score will end up in the database whereas the one with the lower score will be deleted. If two identifications do not agree on the sequence and their scores are similar (given the same software), they are both deleted. In the future, the one with the better internal score will be kept.

### Import Formats

Multiple file formats can be imported to the database. Sequest and GPF file formats are historical. The new file format AMS is more generic and can handle more different types of input. It is to be preferred. In any of the formats the values are to be delimited by one of the following characters: “!Î|”.

#### Sequest File Format

Below is the information that must be present in a file which can be imported to the database. Not all information needs to be given, but the dataset will be more valuable if it is.

Sequest significants file

Name (text [200]) Name of the spot

Dta_file (text [50]) Dta file name

Database (text [200]) Name of the database

Charge (integer) Charge of the precursor ion

Peptide (text [50]) Sequence of the peptide

Mass (decimal) Mass of the precursor ion

Position (integer) Position in the sequest out file

DeltaCn (decimal) Sequest delta Cn value

XCorr (decimal) Sequest XCorr value

DeltaM (decimal) Difference in measured and calculated mass.

Ion_Count (text [10]) Ions found over total ions.

TIC (decimal) Sum over all ions in the dta file.

Reference (text [255]) Database header e.g.: “>scaffold_10”

#### GPF File Format

To import GPF results confirm the format of the text files containing the results first. It has to have the following entries some of which can be left empty:

GPF input file

Base_Name (Text) Usually spot name

Calc_Mass (double) Mass as calculated by GPF

Charge (long) Precursor ion charge

Database (text) Fasta database used for this search

DeltaM (double) Sequest result delta M

Dta_File (text) Name of dta file

GPF_RR (double) Linear regression score from GPF

GPF_Score (double) Tag score from GPF

GPF_XCorr (double) Cross correlation score from GPF

Intron (Boolean) Intron or not

Ion_Count (long) Number of peaks in the spectrum

Left_Fragment (text) Sequence of the part before the intron

Left_RF (long) Reding frame of the left fragment

Left_Start (long) Start in the genome

Meas_Mass (double) Mass of the precursor ion

Peptide (text) Sequence of the peptide

Query (text) De novo prediction

Reference (text) See explanation below

Right_Fragment (text) Sequence of the fragment after the intron

Right_RF (long) Reading frame of the right fragment

Right_Start (long) Start in the genome

Scaffold (text) Database header (here scaffold)

Seq_dCN (double) Sequest delta CN score

Seq_Pos (long) Position in the Sequest out file

Seq_XCorr (double) Sequest XCorr value

TIC (double) Sum of all the intensities in the spectrum

Search_String (Memo) Sequence from the stop up-stream of the left to stop
 downstream of the right fragment

##### Reference Naming Convention

The reference defines the position of the left and right fragment of the split peptide in the genomic database. This position is relative to a sub section of the database. These subsets are called scaffolds in the genomic database of Chlamydomonas reinhardtii. The reference is build of several components:

**%database-header%_%Start-of-left-fragment%_%End%_%Start-of-right-fragment%_%end%**

A string enclosed in % signs should be substituted by a logical value. %database-header% could in reality look like “>scaffold_12”. A typical reference entry would be:

>scaffold_1_10433_10437_11023_11028

#### AMS File Format

One input format contains the following information on one line.

The fields are named as follows (Note that everything would be on one line in for example MS Notepad.

spectrum_id!software!charge!meas_mass!cal_mass!delta_mass!score_1!score_2!score_3!score_4!score_5!sequence_in!sequence_out!left_fragment!right_fragment!left_pos!right_pos!left_rf!right_rf!tic!database!reference!spectrum!search_string!

Following is a brief explanation of the individual fields and what would be expected content.

**spectrum_id**

Here the identifier of the spectrum that was recorded with the mass spectrometer is expected. The format for this identifier needs to adhere to the format described below (Spectrum Naming Format) in short:

%INITIALS%_%GEL%_%SPOT%_%EXPERIMENT%_%MOLMASS%_%PI%.ScanNumber.ScanNumber.Charge.dta

**software**

Here the name of the software that was used for the computational processing of the spectrum is expected. It must be entered as Software in the database with the appropriate thresholds.

**charge**

The charge of the precursor ion that lead to this identification.

**meas_mass**

The measured mass for the precursor ion.

**cal_mass**

The calculated mass from the amino acid sequence assigned to the spectrum.

**delta_mass**

The difference between this two masses (meas_mass – cal_mass)

**scores**

The name of the score followed by a colon and the value that was assigned to this score.

Multiple scores may be separated by commas. E.g.: xcorr:4.56,deltacn:0.12.

The names of the scores must correspond to the names of the thresholds set for this software in the database.

**sequence_in (optional)**

This is the sequence that may have been assigned to the spectrum for example by de novo sequencing.

**sequence_out**

This is the final sequence that has been assigned to the spectrum be the software used in this step.

**left_fragment (optional)**

If the sequence is split into to pieces e.g. spans an intron on the genomic level, the N-terminal fragment should be entered here.

**right_fragment (optional)**

The C-Terminal fragment should be entered here.

**left_pos (optional)**

The start of the left sequence in the genome.

**right_pos (optional)**

The start of the right sequence in the genome

**left_rf (optional)**

The reading frame of the left sequence

**right_rf (optional)**

The reading frame of the right sequence

**tic (optional)**

The total ion current for the MS/MS spectrum

**database (optional)**

The name of the database which lead to the identification

**reference (optional)**

The reference within the database where the identification originates from e.g. Scaffold_1.

**spectrum (optional)**

The spectrum that was recorded by the mass spectrometer. Mass and intensity are separated by commas and multiple values are separated by semi colons. The first two values are precursor mass and charge followed by the spectrum.

**search_string (optional)**

This string includes the peptide sequence and extends it to the closest upstream and downstream stop positions, along the corresponding reading frame. This is the open reading frame.

### Spectrum Naming Format

The full format may look like this:

%INITIALS%_%GEL%_%SPOT%_%EXPERIMENT%_%MOLMASS%_%PI%.ScanNumber.ScanNumber.Charge.dta

%INITIALS% should be user initials,

%GEL% is the name of the gel or separation in general (no underscores allowed within).

%SPOT% is the name of the spot, band or fraction (no underscores allowed within).

%EXPERIMENT% describes how the spot was processed differentially if it was, that is.

It may also merely contain the date or some other identifier.

%MOLMASS% The molecular mass of the spot or band, if known.

%PI% The isoelectric-point of the spot, if known.

It is not allowed to use underscores for any of the variables explained above. These are only used to separate the variables from each other, so that it becomes feasible to automatically assign the results to the correct biological context. Dots are also prohibited from use in the variables since they are automatically added to the name of the dta-files which are created from raw-files for example. These dots are used to automatically determine scan numbers and charge. An example that uses correct naming could be the following:

ja_G1Cab_b13f_2ndM_10000_5.37.1001.1003.2.dta

It is possible to leave out information if it is unknown. Information can only be left out starting from the right. So if the molecular weight is not known you may not enter the pI, either, since it is right of the molecular mass.

Some correct names are shown below:

ja_G1Cab_b13f_2ndM_10000.1001.1003.2.dta

ja_G1Cab_b13f_2ndM.1001.1003.2.dta

ja_G1Cab_b13f_07042006.1001.1003.2.dta

Not correct are the following:

ja_G1Cab_b13f_2ndM_5.37.1001.1003.2.dta missing mol mass

ja_G1Cab_2ndM_10000_5.37.1001.1003.2.dta missing %experiment%

ja_ b13f_2ndM_10000_5.37.1001.1003.2.dta space before b13f and missing %experiment%

They will produce undesired results. The data in the database will definitively be corrupted thus rendering the database unusable.

### DB-Maintenance


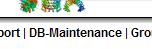
This area can be managed by database administrators. It is located in the Administration area under the Section DB-Maintenance.

#### Problem Data


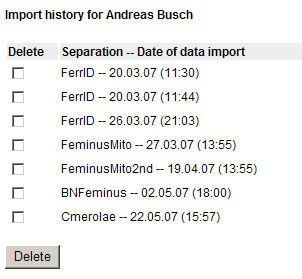
For some reason or other it may be important to remove a dataset from the database at one point. To ensure this an import history is maintained, giving the data that has been imported per user over time. This log may look like the import log on the right. This log is displayed if a user has been selected from the previous menu as described elsewhere in this text. The checkboxes next to the log can be checked and all the rows that are checked will be deleted along with all the data that was contained therein upon clicking delete. This is a function that may be useful if there was some problem with the data file that was imported. It may have been corrupted for example. Then it is essential that the data can be removed from the database again.

#### Database Dump

In addition to deleting problematic imports, it is also possible to save the current state of the database. This is done by following the hyperlink “Make dump”. Upon clicking, the dump is created which may take a while, depending on the size of your database.


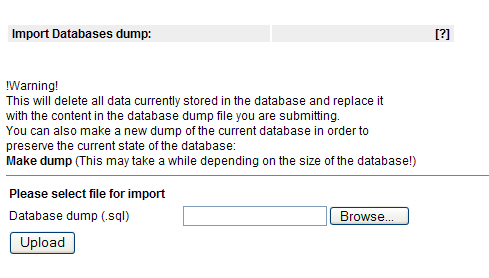


Once the dump has been created you are able to download it. This dump may be used to reestablish the current state in a new database. Or maybe you move to a new server and you don’t want to import everything and setup everything again. In these cases a dump comes in very handy.

### Software


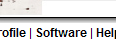
***General***

Users are not able to make any changes.

***For Administrators***

If you want to import mass-spec data to your database you have to make sure that the settings you made in your analysis Software like Sequest, also exist in your database. If thresholds are missing we can not assure that the data will be imported correctly.

Only Administrators are able to create, delete and update thresholds for software. They can define the default settings for the analysis software used for the mass-spec data.

Each threshold is represented by a row and contains one parameter minimum. A parameter contains three settings: name, operator (>, <, >=, <=, = and ≠) and value.

(Figure …)


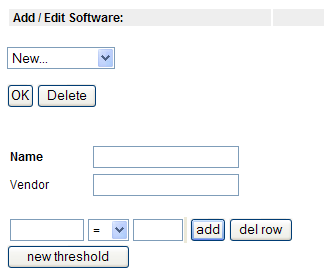


With the “New Threshold”-Button you can add a new threshold which appears as a new row, with a single parameter. With the “add”-Button you can add a new parameter to your thresholds. The amount of parameters can differ between the different thresholds. With the “delete”-Button you delete the whole Threshold with all its parameters. Deleting a single parameter is not possible at the moment!

If you just want to change the values for the parameters, the software name, the operators, etc. just click on “Confirm Changes” after you made your settings.

After you made all your changes click on the “Apply Changes to Users”-Button, so all users get the new software settings. All settings made by users for their software thresholds are overwritten, so inform your users, if you make an update!

###
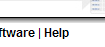
Help

The data contained under help is similar to the data contained in this manual. The difference is that the help file is without images due to the fact that it is context sensitive, thus delivering help to the area that is needed at that moment in time.

# General Area

## Home

This area is merely a welcoming screen for the database application which does not provide further functionality.

## Experiments


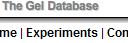
This section gives a preview for all experiments that are contained in the database.

The number of experiments is increasing over time which is why a filter can be applied such that not all experiments are displayed in this preview section. The values that can be filtered are shown in the table below:

| Filter | Result |
| --- | --- |
| author: | Filters for the author of the gel who is the person that stored the gel in the database not necessarily the first author of the associated publication. |
| journal: | Filters for the name of the journal. |

The filters can be entered into the field and following the filter a criterion should be provided (author:kuhl). The criterion does not have to be provided completely. In the example all experiments made by Kuhlgert would be displayed.


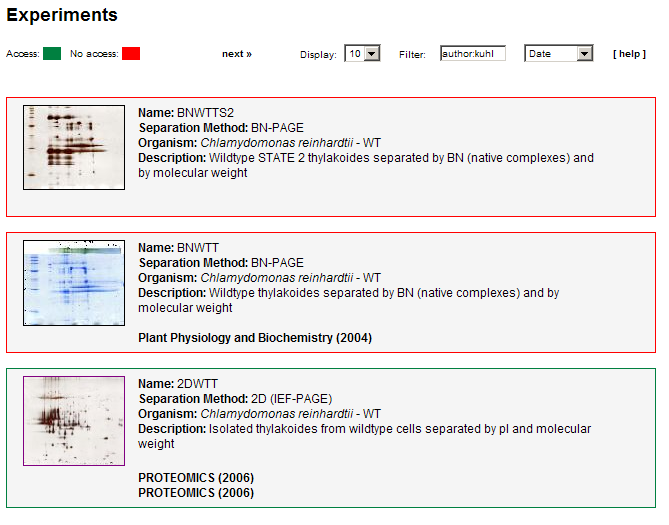


Figure 7: Typing journal:prot would return all experiments with a publication in a journal containing for example proteomics in the name; journal:omics would even return genomics and other possible hits. However the number of distinct journals and distinct experiments is probably not very strongly correlated. The results can further be sorted by different criterions in the drop-down box next to the filter field. Generally the experiment details that are accessible are framed in green whereas the results that may not be accessed with the current login are presented in red.

## Compare


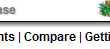
In order to access this area you need to be logged in with sufficient rights. If you are, you may choose 2 images of your experiments. This are then scaled and displayed next to each other. This may be useful in order to assess the similarity of two for example 2D gels. In general, there are specialized software tools, which will help you a lot more with this, but having a quick glance can sometimes answer a lot of questions. For this reason we provided this feature.


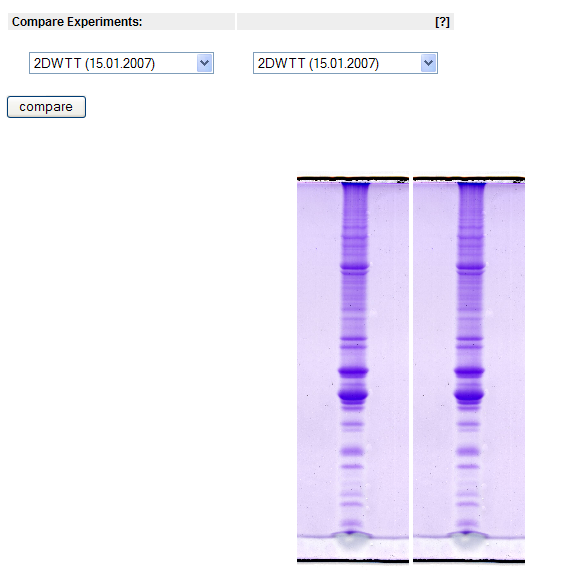


Figure 8: here are two distinct one dimensional gel electrophoresises which can be compared visually using this tool. Some differences are visible even at this low resolution, which is of course better on the web.

## Getting Started


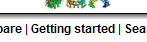
Here some information is contained which will since you have the manual not be interesting to you since it is redundant with the information presented here.

##
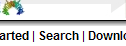
Search

Database usually contain huge a amount of information. In proteomics database there are also different types of information contained. This information may contain interesting patterns that need to be identified. One example would be the location of a protein during multiple experiments. Using the search facility which is provided with 2DB it is easy to find a wealth of interesting information.


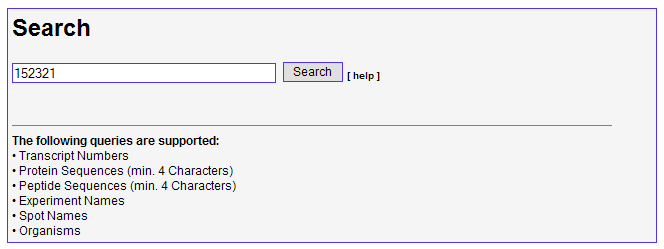


Figure 9

The search facility offers a field which accepts a free text search. The search only accepts one sequence of alpha numeric characters at the moment. The underscore character “_” can be used as a wildcard for one missing character (e.g.: 2_B would find 2DB). The percent sign “%” can be used for longer sequences that are missing (e.g.: A%er would find Allmer). The search will return proteins, if protein identifiers are entered. It will return sequences and peptides if sequences are entered. The locations that these peptides and proteins have on the gels are given in the results as well.

All this information is displayed in respect to the rights of the user. Confidential data that has not been opened to public will not be displayed to the public, but to a member of the research lab it may be available.


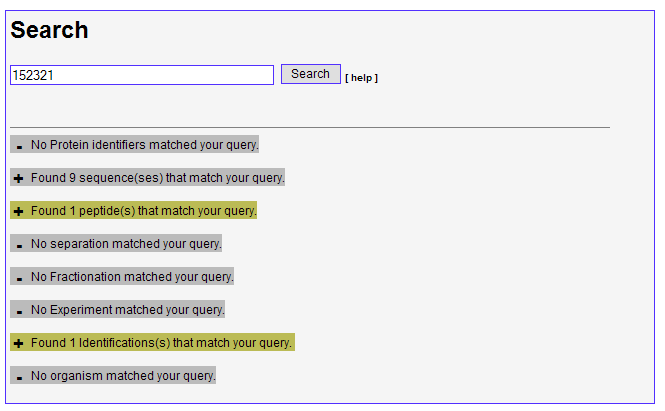


Figure 10:Here is the result of a search with the 2DB application. Some of the possible result types such as proteins, sequences, organisms and so forth did yield results for the query. Some of the possible types didn’t. Note that in any instance of 2DB only positive results are returned whereas other types are not listed.

After the search is submitted a result list as displayed in Figure 10 displays the areas where matches were found. Each of these areas typically contain many matches, depending on the query and the size of the database.


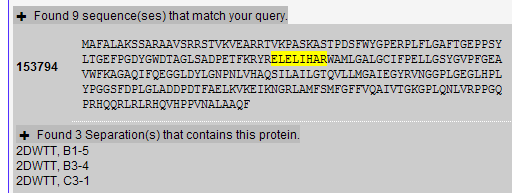


Figure 11: Here a section of the sequences that were found is shown. First the sequence is shown with the matching query highlighted. Then the matches in the accessible experiments are shown. First the image of the experiments is shown as a thumbnail and then all hot spots on the image map which identified that sequence are given as hyperlinks to the detailed results.

They contain additional/ more detailed information, as can be seen in Figure 11. It shows not only the sequence with the match highlighted within the sequences, but also all available identifiers and aliases as hyperlinks left of the sequence. The plugins which work with sequences are also available in this context. If the protein or sequence has been encountered in any experiment that the current user is eligible to view, these experiments are listed below the sequence along with a thumbnail and hyperlinks to access detailed information about the identifications.

## Download


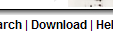
This section will show where to find a download for 2DB. If a new version is available and you are logged in as an administrator, you can also update your current version to the new version. This is highly recommended, since new features may be extremely useful. An upgrade will never compromise your database in a way that you would have to reinstall it. If in doubt, dump your data first (Administration – Import – Create Dump) and then upgrade to the new version.

## Help


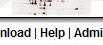
The data contained under help is similar to the data contained in this manual. The difference is that the help file is without images due to the fact that it is context sensitive, thus delivering help to the area that is needed at that moment in time.

## Administration


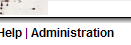
If you have been authenticated as an eligible user of this instance of 2DB, and your rights are sufficient, a new link is displayed on the right. This link will allow you to access the administration area. Obviously, you may also type in the page you would like to access in the administration area directly, but that will just transfer you back to the welcoming page if you haven’t been properly authenticated with an encrypted cookie.
